# Energetics and mechanism of anion permeation across formate-nitrite transporters

**DOI:** 10.1038/s41598-017-11437-0

**Published:** 2017-09-20

**Authors:** Kalina Atkovska, Jochen S. Hub

**Affiliations:** 1 University of Goettingen, Institute for Microbiology and Genetics, Goettingen, 37077 Germany; 2 University of Goettingen, Göttingen Center for Molecular Biosciences, Goettingen, 37077 Germany

## Abstract

Formate-nitrite transporters (FNTs) facilitate the translocation of monovalent polyatomic anions, such as formate and nitrite, across biological membranes. FNTs are widely distributed among pathogenic bacteria and eukaryotic parasites, but they lack human homologues, making them attractive drug targets. The mechanisms and energetics involved in anion permeation across the FNTs have remained largely unclear. Both, channel and transporter mode of function have been proposed, with strong indication of proton coupling to the permeation process. We combine molecular dynamics simulations, quantum mechanical calculations, and p*K*_a_ calculations, to compute the energetics of the complete permeation cycle of an FNT. We find that anions as such, are not able to traverse the FNT pore. Instead, anion binding into the pore is energetically coupled to protonation of a centrally located histidine. In turn, the histidine can protonate the permeating anion, thereby enabling its release. Such mechanism can accommodate the functional diversity among the FNTs, as it may facilitate both, export and import of substrates, with or without proton co-transport. The mechanism excludes proton leakage via the Grotthuss mechanism, and it rationalises the selectivity for weak acids.

## Introduction

Formate-nitrite transporters constitute an ancient family of transmembrane proteins involved in the translocation of monovalent anions across biological membranes^1,2^. FNTs have so far been linked to transport of formate, nitrite, hydrosulfide, lactate, acetate, and bicarbonate^3–11^. These proteins have been found in bacteria, archaea, and unicellular eukaryotes, but not in higher organisms^12^. The nitrite channel NirC has been implicated in the pathogenesis of *Salmonella typhimurium* ^13^ and of the avian pathogenic *Echerichia coli* ^14^, while the *Plasmodium* lactate transporter *Pf*FNT has been suggested as an antimalarial drug target^9,15,16^.

**Figure 1:**
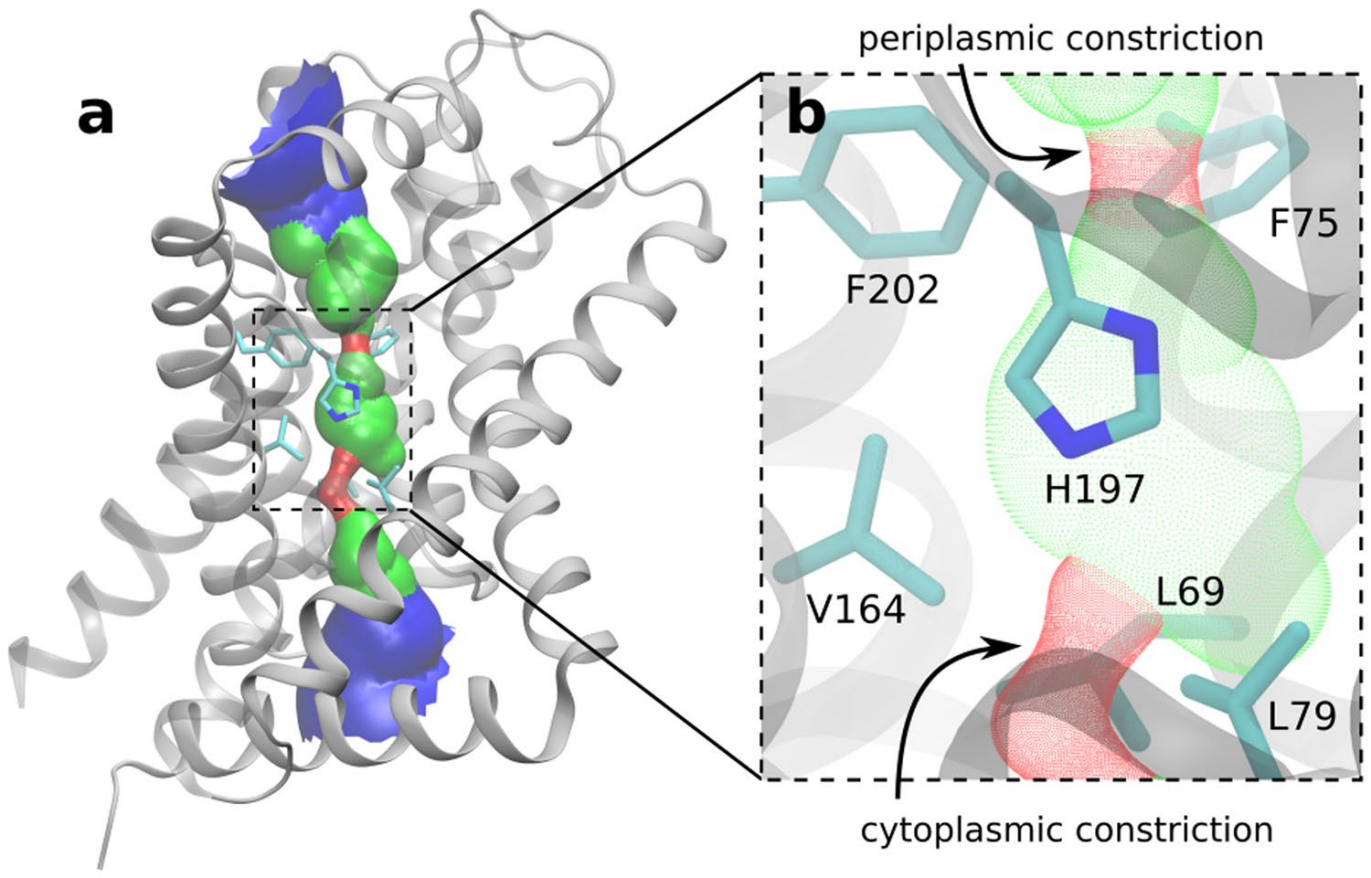
Crystal structure of a NirC monomer (PDB ID: 4FC4). The side chains of the constriction-forming residues and of the central histidine are shown as sticks and labeled in the zoomed image. Pore representation done with HOLE^77^, colour coded by pore radius: red < 1 Å < green < 2.5 Å < blue.

Three prokaryotic FNT subfamilies have been characterised: NirC, a channel essential for nitrite uptake^5,17–19^; FocA, a bidirectional formate channel^3,20–22^; and HSC (also known as FNT3 or AsrD), a hydrosulfide channel necessary for substrate export^8^. Available crystal structures for five representatives from these subfamilies reveal a homopentameric architecture with a five-fold symmetry axis perpendicular to the membrane, and a remarkable structural similarity with the water channels aquaporins^8,19,23–25^. Each monomer is a twisted bundle of six transmembrane helices, traversed by the narrow permeation pore that is connected to the cytoplasmic and periplasmic space via two funnel-shaped entrances (Fig. 1a). Two constriction sites made up of hydrophobic residues delimit a central chamber, where a highly conserved histidine residue (hereinafter “the central histidine”) represents the only polar residue (Fig. 1b). The region connecting subhelix TM2a to helix TM3 (topology nomenclature as in ref.^23^), was resolved in two different orientations in the *Vc*FocA structure and named the “Ω-loop”^24^. In contrast, this region exhibits a well-defined helical structure in *Ec*FocA, NirC, and HSC^8,19,23^ (Supplementary Fig. S1a–d).

The term “transporters” for these permeases originates from an early classification of transport proteins derived from genome analyses^26^, and was based on a putative acetate/H^+^ symporter in yeast^10^. Previously, FocA was described *in vivo* as a bidirectional formate channel, without excluding the possibility for formate/H^+^ symport^3^. The discovery of the aquaporin-similar structure pointed towards a channel-like permeation mechanism, a view supported by two electrophysiological studies of NirC and FocA, which demonstrated voltage-independent anion transport with a conductance of ~25 pS, and polyspecificity for a range of monovalent anions^19,27^. A nitrite/H^+^ antiport activity of NirC has also been suggested^28^. Most recently, the plasmodial transporter *Pf*FNT was reported to act as a lactate/H^+^ symporter^9,29^.

Previous data have not provided a consensus on many mechanistic aspects of the permeation across the FNTs, including the roles of the central histidine, the Ω-loop, and of an involved proton. In addition, the mode of permeation as channel or symporter has remained obscure. The central histidine has repeatedly been suggested as crucial for permeation, possibly through its side-chain orientation^23^, or by interactions with the permeating substrate^1,24^ and by substrate protonation^2,19^. Indeed, mutation of this histidine resulted in no measurable current in FocA^27^. The conformation of the Ω-loop in *Vc*FocA has been proposed to play a role in channel gating^24^. The FNT family has been suggested to contain both, channels and transporters, similar to the ClC family for chloride transport^1^. Alternatively, a “proton relay” mechanism has also been proposed, in which the central histidine cycles between its neutral (HIS0) and positively-charged (HIS+) form, while transiently protonating the permeating ion, thereby enabling it to pass through the hydrophobic constrictions^2,19^. A different substrate protonation mechanism occurring in the FNT entrances and involving a shift in the substrate acidity, has been recently described^30^.

We present a comparative analysis of the energetics of anion permeation across all FNT subfamilies with a known structure, with or without proton co-transport. We demonstrate the functional roles of the proton and the central histidine in the permeation process, as well as the importance of the conformation of the Ω-loop in FocA. To this end, we used molecular dynamics (MD) simulations to calculate potentials of mean force (PMFs, sometimes referred to as “free energy profiles”) for permeation across the FNTs, while considering different protonation states of the central histidine and permeating substrate. The simulations suggest a necessity for substrate protonation in order to complete the permeation. This mechanism was further investigated in NirC, for which we performed additional sets of free energy calculations and quantum mechanics/molecular mechanics (QM/MM) simulations, revealing a quantitative picture of anion permeation across the FNTs.

## Results

**Figure 2:**
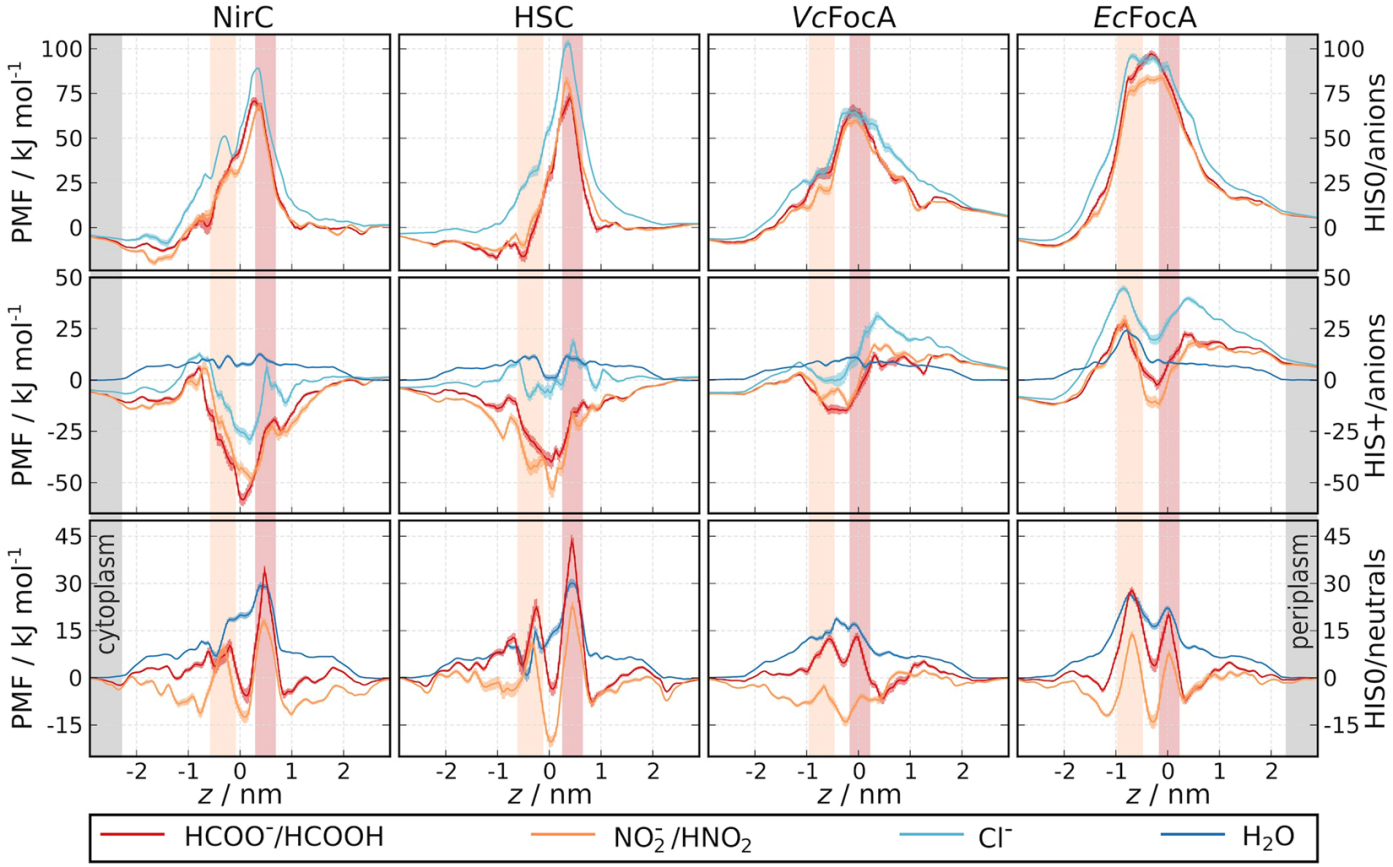
PMFs for permeation of different substrates (see legend) across NirC, HSC, *Vc*FocA, and *Ec*FocA (from left to right), calculated using umbrella sampling. Top row: permeation of anions across a pore with a neutral central histidine (HIS0), middle row: permeation of anions and water across a pore with a positively-charged central histidine (HIS+), bottom row: permeation of neutral substrates and water across a HIS0 pore. The tan and brown bars indicate the cytoplasmic and periplasmic constriction, respectively. Anions experience a high barrier for permeation across the HIS0 pore, and strong binding into the HIS+ pore.

In order to determine the energetics of permeation across the FNTs, we calculated PMFs for permeation of multiple substrates across NirC, HSC, *Vc*FocA, and *Ec*FocA, considering all plausible combinations for the protonation states of the central histidine and the permeating substrate. Figure 2 presents the PMFs as a function of the pore coordinate *z*, where *z* = 0 corresponds to the centre of mass of the transmembrane parts of the pentamer. For illustration, representative snapshots from the simulations corresponding to the minima in the PMFs for permeation of neutral or ionic substrates across a HIS0 or HIS+ pore, respectively, are shown in Supplementary Fig. S2.

### Anions do not permeate the FNT pore

All tested anions encounter a very high permeation barrier of 60 to 105 kJ mol^−1^ when the central histidine is in its neutral form (Fig. 2, top row), which is in good agreement with previous calculations involving formate permeation across *Vc*FocA^31^. The large barrier reflects that an anion would lose its hydration shell upon permeation, and that the hydrophobic channel does not provide adequate compensating interactions. The barriers for all anions would practically result in no permeation across the FNTs, which is consistent with a permeation mechanism that necessitates a proton involvement.

The permeation barriers for the neutral counterparts of the anionic substrates across a HIS0 FNT pore are significantly lower (Fig. 2, bottom row). However, with respective p*K*_a_ values of ~3.8 and ~3.3, formic and nitrous acid are not expected to significantly contribute to the substrate permeation in their neutral form at most physiological conditions. Moreover, a permeation of a neutral substrate would generate no signal in electrophysiological studies. Hence, the functional mechanism of the FNTs cannot be fully explained by a neutral substrate permeation.

The PMFs for anions drastically change upon protonation of the central histidine (Fig. 2, middle row). Now, the anions strongly bind to the imidazolium ring, with binding free energy for formate and nitrite in the range of −60 to −30 kJ mol^−1^ in NirC and HSC, and −15 kJ mol^−1^ in *Vc*FocA. Moreover, the barrier for ion internalisation into the pore is nearly eliminated in NirC, HSC, and *Vc*FocA. Chloride ions also bind in the central chamber in all FNTs, albeit significantly weaker than formate and nitrite, suggesting the FNTs are specialised to form favourable interactions with their functionally relevant anions.

When comparing the PMFs for permeation across the FocA HIS+ pores, we register a drop in the peak in the region of the Ω-loop and the cytoplasmic constriction in *Vc*FocA, as compared to *Ec*FocA (Fig. 2, middle row *z* ≈ −1). In simulation, the Ω-loop in *Vc*FocA demonstrated a significantly higher structural variability among monomers and in time as compared to *Ec*FocA (Supplementary Fig. S1e–h), emphasising the role of the conformation of the Ω-loop in anion permeation across the cytoplasmic constriction in FocA. In NirC and HSC, on the other hand, the stable helical structure of the same region does not seem to pose an obstacle for permeation (no significant peak at *z* ≈ −0.5 in Fig. 2, middle row).

The strong binding of formate and nitrite to the HIS+ pores implies that the anion alone cannot complete the permeation with the experimentally observed rates. To test if a knock-on mechanism yields an efficient anion translocation across the FNTs^31^, we performed extensive computational electrophysiology (CompEl) simulations of NirC and *Vc*FocA in the HIS+ state^32^. In those simulations, anions rapidly entered the channel, as expected from the low internalisation barriers in the PMFs (Supplementary Fig. S3). However, even at saturating salt concentrations and high transmembrane potentials (0.6–1.0 V) only very few permeation events were observed, translating into insignificant conductances of 0–0.1 pS (Supplementary Table S1). Hence, the CompEl simulations suggest that a knock-on mechanism does not apply in the FNTs.

Taken together, the PMFs and CompEl simulations imply that anions are not able to completely traverse the FNT pore, regardless of the protonation state of the central histidine. This is either due to a high free energy barrier for permeation in the HIS0 pore, or due to strong binding to the central histidine in the HIS+ pore. This supports the view that anion permeation across the FNTs is achieved by substrate protonation, the details of which we studied further in NirC. Considerable amount of experimental work has been done to characterise NirC^5,17–19^, however, to the best of our knowledge, there are no computational studies describing the energetics of permeation across this protein with molecular explanation. Moreover, crystallography data and our simulations reveal a stable region of the Ω-loop in NirC, which reduces the complexity of the system when studying the steps of anion permeation across the FNT pore.

### Permeation mechanism in NirC

Given that anions would only very rarely enter a HIS0 pore, but are rapidly internalised into a HIS+ pore, we aimed to quantify the feasibility of reaching a HIS+ state in the NirC pore, by computing the free energy of protonation of the central histidine. We further performed QM/MM simulations in order to investigate the possibility of anion protonation by the central histidine.

#### The central histidine is neutral in absence of an anion

Considering the central histidine is located deep within a hydrophobic pore, protonating this residue may be associated with a high cost in free energy. Here, we calculated the free energy of protonation Δ*G*_prot_ of the central histidine using a combined molecular mechanics/continuum electrostatics approach, as implemented in the generalised Monte Carlo titration (GMCT) method^33^. In absence of a proton motive force, we found Δ*G*_prot_ ≈ 70 kJ mol^−1^ (Supplementary Table S2), which is reduced by only 10 kJ mol^−1^ at a proton motive force of −170 mV (Supplementary Fig. S4). Such a high free energy cost of protonation would result in a negative p*K*_a_ and extremely low protonation probability at physiological conditions, as long as no anions enter the pore. We also tested the effect of the dielectric constants of the protein and the protein cavities on the calculated Δ*G*_prot_ (Supplementary Table S2), showing that even with a high protein dielectric constant, the protonation of the central histidine is highly unfavourable. Moreover, these findings were qualitatively confirmed by MD simulations using thermodynamic integration (see Methods).

A low protonation probability for the central histidine is compatible with a stop-flow experiment that detected no water permeation across FocA^23^. Indeed, in HIS+ simulations, we observed water-filled pores and a low water permeation barrier of ~12 kJ mol^−1^, close to the barrier of 13 kJ mol^−1^ reported for aquaporins^34^, suggesting that the HIS+ pore would be a water channel (Fig. 2, dark blue curves, middle row). In contrast, the PMFs for water permeation across a HIS0 pore reveal barriers of ~20–30 kJ mol^−1^, characterising a poor water channel (Fig. 2, dark blue curves, bottom row), in line with the stop-flow experiment. In this way, maintaining a low protonation probability of the central histidine may be a strategy to exclude proton leakage via the Grotthuss mechanism.

#### Mutual stabilisation of anion and proton into the pore

**Figure 3:**
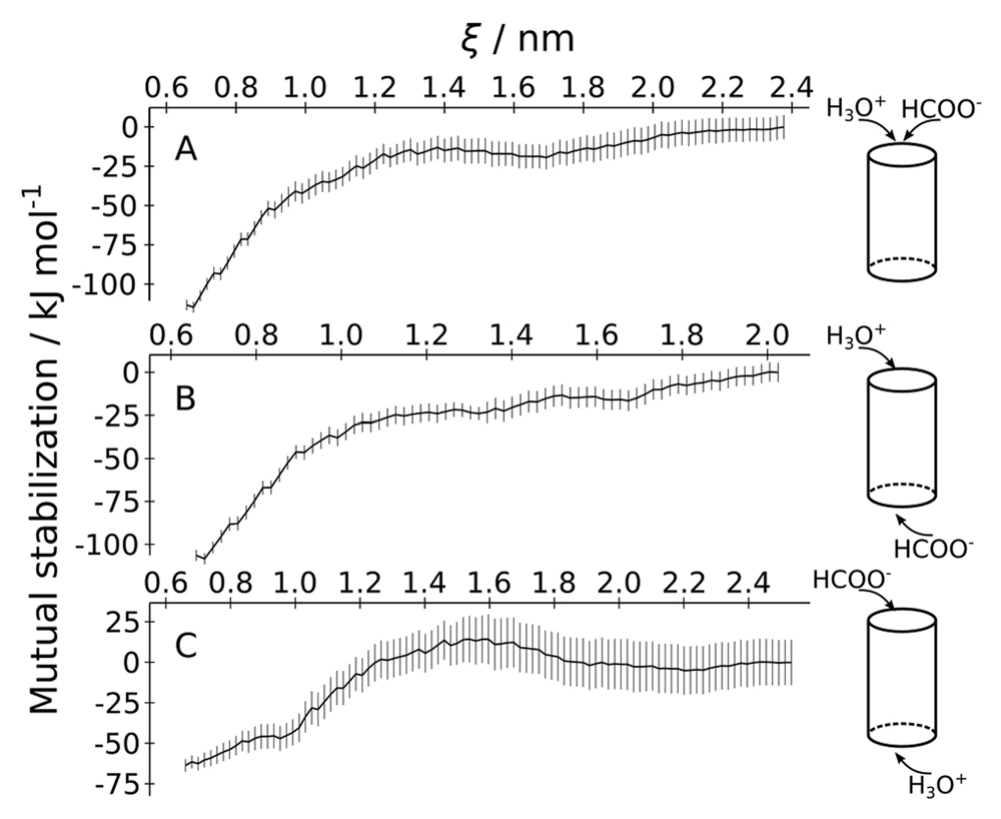
Mutual stabilisation of a formate and hydronium ion entering the pore. The curves denote the difference between (i) the sum-of-distances PMF for simultaneous internalisation of the ions Δ*G*_sum_(*ξ*), and (ii) the sum of the single-ion PMFs of formate and a classical hydronium model, while considering different compartments of origin of the ions: (**A**) both ions enter from the periplasmic space, (**B**) the hydronium and formate ion enter from the periplasmic and cytoplasmic space, respectively, (**C**) the hydronium and formate ion enter from the cytoplasmic and periplasmic space, respectively.

The PMFs for anion permeation reveal a strong favourable interaction (~−60 kJ mol^−1^) of anions with the positively-charged central histidine in the NirC pore (Fig. 2, middle row). Given the similar free energy cost for protonation of this residue (~60–70 kJ mol^−1^), we hypothesised that coupling of the processes of histidine protonation and anion binding result in a thermodynamically accessible state. In other words, we hypothesised that *simultaneous* internalisation of proton and anion into the NirC pore may mutually stabilise the ions. A rigorous free energy calculation of such simultaneous internalisation aiming at a two-dimensional PMF, possibly using physically accurate delocalized proton models, would be computationally highly demanding. Therefore, in this work, we do not try to describe the exact proton pathway and complete free energy landscape of simultaneous internalisation, but we instead took a semi-quantitative approach: we calculated the “sum-of-distances” PMFs Δ*G*_sum_(*ξ*), where the reaction coordinate *ξ* was defined as the sum of the formate-HIS0 and hydronium-HIS0 distances (Supplementary Fig. S5a), considering different combinations of the direction of entrance of the ions: (a) hydronium and formate from the periplasmic side; (b) hydronium from the periplasmic, formate from the cytoplasmic side; and (c) formate from the periplasmic, hydronium from the cytoplasmic side (Supplementary Fig. S5c). To quantify the “mutual stabilisation” of the ions (Fig. 3), we calculated the difference between (i) Δ*G*_sum_(*ξ*), and (ii) the sum of the single-ion permeation PMFs for formate and hydronium (see Methods for details). Due to the classical description of the hydronium used here, these calculations can not reveal the exact migration pathway of the proton, but they provide an order-of-magnitude estimate for the favourable anion-proton interactions in the pore, as compared to introducing each of these ions in the pore alone.

Figure 3 demonstrates that indeed, a proton and an anion could stabilise each other in the pore, and this effect could be in the order of tens of kJ mol^−1^ close to the central histidine (*ξ* ≈ 0.6 nm). We observe more pronounced effect in cases where the hydronium ion enters from the periplasmic space, which corresponds to the physiological proton gradient over the bacterial inner membrane (Fig. 3A,B). The distributions of the individual formate-HIS0 and hydronium-HIS0 distances extracted from the umbrella sampling simulations used for calculation of Δ*G*_sum_(*ξ*) (Supplementary Fig. S5e) suggest that in the hypothesised coupled process, the formate ion would tend to enter the pore vestibule first, only after which the hydronium ion would follow.

In absence of more rigorous proton models, such as reactive or delocalised proton models, the coupling of the histidine protonation and anion binding in the pore cannot be unambiguously and fully quantitatively demonstrated. However, the significant free energy gain evident from the mutual stabilisation calculated here, suggests that proton-anion interactions could be highly relevant for reaching a permeation-competent state of the channel, rendering a coupled penetration of the anion and proton into the pore more likely than penetration of either the proton or the anion alone.

#### Substrate protonation by the central histidine

**Figure 4:**
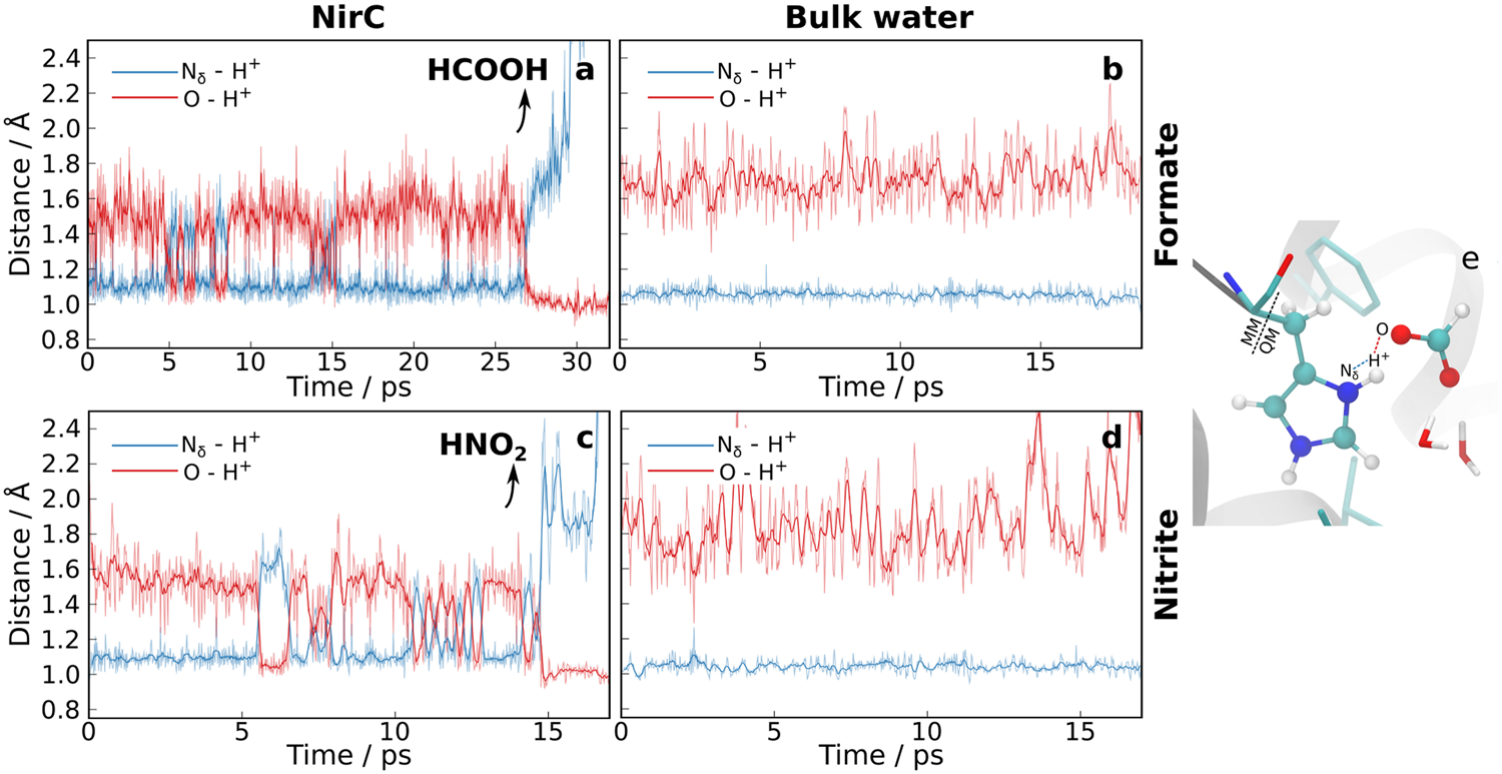
Proton transfer between formate (**a**,**b**) or nitrite (**c**,**d**) and the central histidine in the NirC central chamber (**a**,**c**) or a capped histidine residue in bulk water solution (**b**,**d**). The distances of the proton to the donor atom (N_*δ*_ atom of the histidine), and to the acceptor atom (an oxygen atom of formate or nitrite) are shown vs. time (raw trace and running average). In the protein environment, frequent proton jumps between the central histidine and the bound anion are observed on a picosecond time scale. (**e**) Simulation snapshot from the central chamber, illustrating the distances plotted in the graphs. Atoms of the QM region are shown as spheres.

Finally, once the anion is bound to the HIS+ FNT pore, we investigated whether an anion protonation by the central histidine is possible. To this end, QM/MM simulations of NirC with formate or nitrite bound to this histidine were performed (Fig. 4e). The histidine side chain and the bound anion were described quantum mechanically, while the rest of the system was described classically. The distances of the proton to the donor atom (the N_*δ*_ atom from the central histidine), and the acceptor atom (an oxygen atom from the formate/nitrite ion) are shown in Fig. 4a,c as a function of time. The simulations demonstrate that the proton is able to jump between the histidine and the anion on a picosecond time scale, despite the lower p*K*_a_ of formic acid as compared to histidine. Indeed, we observe proton transfer events between the histidine residue and a formate/nitrite ion only in the protein binding site (Fig. 4a,c and Supplementary Fig. S6a), and not in bulk water (Fig. 4b,d and Supplementary Fig. S6b), suggesting that the more hydrophobic protein environment (as compared to bulk water) favours the neutral over the charged species. In both simulations shown in Fig. 4a,c, the substrates eventually dissociated from the binding site in a protonated form.

## Discussion

This work provides a quantitative comparison of the energetics of permeation across the FNTs, with a special focus on NirC, whose permeation mechanism was studied in detail. In general, we find that anions are not capable of completing a permeation across the FNTs, due to either a high barrier posed by the HIS0 pore, or due to strong binding to the HIS+ pore. Substrate permeation in a neutral form is also unlikely at physiological conditions that favour the ionic species, moreover, such permeation is incompatible with electrophysiology experiments. Taken together, our simulations are consistent with a permeation mechanism that requires anion protonation during permeation^9,19,30^.

We performed multiple sets of free energy calculations and QM/MM simulations, which reveal the following permeation mechanism for nitrite and formate across NirC: (i) the protonation of the central histidine and the translocation of the anion into the pore probably occur in a coupled manner, (ii) the anion is protonated by the central histidine, and (iii) the now neutral substrate faces a weaker binding and may exit the pore.

**Figure 5:**
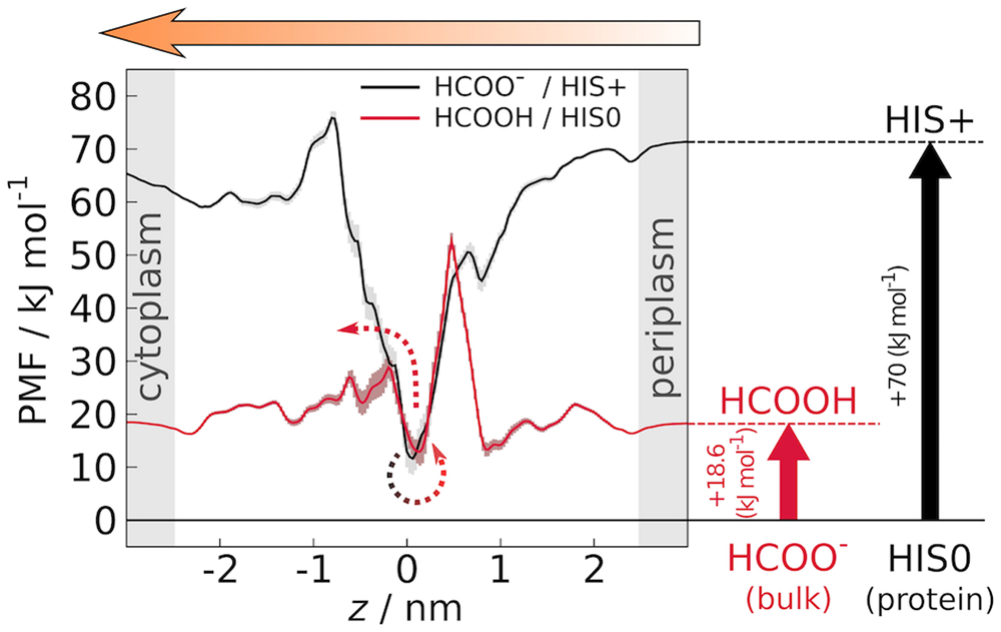
Permeation of formate across NirC. Shown are the PMFs for permeation of formate across a HIS+ pore (black) and for permeation of formic acid across a HIS0 pore (red), corrected for the respective free energies of protonation of the central histidine in the protein (black arrow on the right), and of formate in bulk assuming pH = 7 and taking the formic acid p*K*_*a*_ of 3.75 (red arrow on the right). The arrow on the top denotes the main physiologically-relevant direction of permeation across NirC. Once the anion is bound to the central histidine (*z* ≈ 0 at the black curve), it can be quickly protonated by it (jumping from the black to the red PMF at *z* ≈ 0), after which the substrate is enabled to leave the pore more easily, with lowest barrier towards the cytoplasm.

Anion internalisation in the FNT pore is highly unlikely when the central histidine is neutral, while anions readily enter the HIS+ pore, as observed from the PMF calculations (Fig. 2, middle row) and CompEl simulations (Supplementary Fig. S3). However, the protonation of the central histidine needs to be coupled to anion binding, in order to compensate for the high free energy cost for protonation. The energetics involved in the permeation are summarised in Fig. 5, which depicts the PMFs for permeation of formate across a HIS+ pore (black curve), and formic acid across a HIS0 pore (red curve), corrected for the free energy cost of protonating the central histidine, or protonating the anion in bulk, respectively. Once the anion is bound to the central histidine (*z* ≈ 0 at the black curve), it is not able to complete the permeation unless it is protonated, which can occur on a picosecond timescale, as predicted by the QM/MM calculations. In Fig. 5, this is represented by jumping from the black to the red PMF at *z* ≈ 0. By taking up a proton, the substrate is enabled to leave the pore more easily. The lowest permeation barrier is presented by the cytoplasmic constriction (Fig. 5, red curve), which is in accordance with the role of NirC to import nitrite to the cytoplasm, where it can be further reduced by the cytoplasmic nitrite reductase NirBD^18,19^. Finally, whether the substrate carries the proton up to the bulk solvent, or returns it to the central histidine via a relay mechanism after it crosses the barrier^19^, needs to be further investigated.

How likely is an alternative permeation pathway of a neutral substrate across the HIS0 pore, thus circumventing the histidine protonation step? For NirC, a neutral substrate would face a high permeation barrier posed by the periplasmic constriction (Fig. 5, red curve, *z* ≈ 0.5), rendering such a pathway unlikely. In contrast, this barrier is lower in FocA (Fig. 2), suggesting that the PMF cannot exclude the possibility that formate import in FocA involves a permeation of a neutral substrate across a HIS0 pore, with the protonation step occurring most likely in the hydrophobic vestibule, before crossing any of the constrictions^30^.

The majority of calculations in the present study demonstrate that the pore environment strongly favours neutral over charged species. This is clearly seen in the high free energy barriers for permeation of anions, the high energetic penalty for introducing a positive charge on the central histidine, as well as the fast proton transfer from this histidine to a bound anion. These observations are in accordance with the proposed dielectric shift of the substrate acidity in the hydrophobic environment of the FNT pore, leading to easier substrate protonation^30^.

The hypothesised coupling between (i) the histidine protonation and (ii) the anion binding may act as a type of substrate gating, such that a HIS+ state of the pore is reached only in the presence of anionic substrates. Notably, a similar situation has been shown to apply in the ClC family of chloride channels and exchangers, where the protonation of a gating glutamate side-chain occurs only in the presence of chloride ions^35,36^. In FNTs, the requirement of an anionic substrate in order to achieve histidine protonation may also prevent excessive proton leakage via the Grotthuss mechanism, as water is efficiently translocated only by the HIS+ pore, and not by the HIS0 pore. The requirement for substrate protonation in turn, makes the channel selective for weak acids, and prevents leakage of other anions, such as chloride. Indeed, in electrophysiology experiments, chloride ions were shown to bind, but not to efficiently permeate the FNTs^27^.

**Figure 6:**
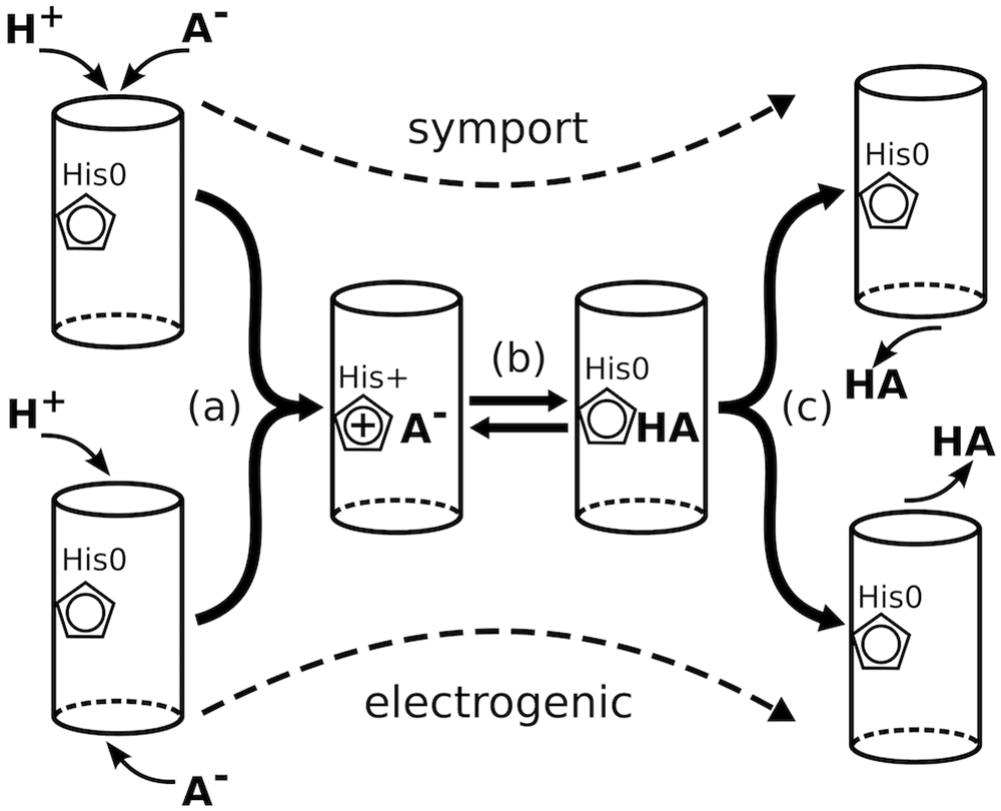
Permeation mechanism including (**a**) simultaneous internalisation of anion (A^−^) and proton (H^+^) into the pore, (**b**) anion protonation by the central histidine into its neutral counterpart (HA), and (**c**) release of the neutral substrate from the pore. This mechanism allows for anion import or export in an electrogenic channel-like manner (bottom branch) or as proton symport (top branch).

As previously stated, members of the FNT family have been found to be relevant for import, export, or both import and export of anions, and have been suggested to perform this function with proton co-transport, via a proton relay mechanism, or as simple channels^1,2,30^. This functional diversity within the family can be accommodated by the permeation mechanism by substrate protonation described above, since in principle it allows for a bidirectional transport in both channel-like and proton-symport manner (Fig. 6). Channel-like electrogenic transport would be achieved when the anion and proton originate from opposite sides of the membrane, otherwise, the anion and proton would be symported. This “adaptive” property of the proposed mechanism can potentially account for the cessation of the electrogenic current and increase of the substrate/proton symport at acidic pH^9,27,29^. Naturally, not all directions and modes of permeation need to be present and feasible for each FNT protein, and will depend on the given physiological conditions. In NirC, the channel-like mode is relevant in electrophysiology experiments, where the Nernstian behaviour of the current suggests specific anion transport^19^. However, at physiological conditions, both protons and nitrite ions have inward electrochemical gradients (assuming high activity of NirBD in the cytoplasm), and would likely tend to enter the pore from the periplasmic side, resulting in an anion/H^+^ symport. The report of *in vitro* proton-antiport activity of NirC^28^ is not in line with the mechanism proposed here. However, as elaborated in ref.^19^, such activity would be unlikely in physiological context, since it would entail depriving the cytoplasmic nitrite reductase NirBD (co-expressed with NirC) from its substrate.

The primary role of the proton in the permeation process is to modify the free energy profile along the pore, in order to enable efficient anion transport. By protonation of the central histidine, anion internalisation into the centre of the pore is allowed, while the protonation of the substrate enables its release from the strong binding. Additionally, the proton could be used to drive the anion transport across the membrane, if necessary. In the case of NirC, in presence of a sufficient nitrite inward concentration gradient, electrogenic nitrite import is thermodynamically possible, however, proton symport is more favourable due to the negative electric potential across the membrane.

An inspection of the PMFs reveals higher similarities between NirC and HSC on one side, and the FocA channels on the other, which is consistent with their phylogenetic relationship and structural similarity^2^. In NirC and HSC, the region of the Ω-loop was not found to be a major obstacle for permeation. In contrast, a major difference between the two investigated FocA proteins is observed in this region, where higher level of structural variability resulted in a lower permeation barrier for both, anions and neutral substrates (Fig. 2, third and fourth column, *z* ≈ −1). The higher peaks in this region of the PMFs for permeation across *Ec*FocA suggests that the *Ec*FocA crystal structure might indeed represent a closed state, and that we do not sample opening transition in *Ec*FocA in our simulations. This is not the case in NirC, where no experimental evidence of such transition has been found, and simple side-chain fluctuations on substrate permeation have been proposed to be sufficient for channel function^19^. The orientation of the Ω-loop in FocA may be tightly connected to the structure of the flexible N-termini^25^ and their function in binding formate-producing enzymes to the cytoplasmic surface of FocA^37,38^, suggesting that permeation across FocA, including the role of the Ω-loop, have to be considered in a wider metabolic context.

In conclusion, our results characterise FNTs as a striking example for a protein family existing on the border between channels and transporters. We demonstrate how a tightly controlled protonation event may (i) constitute the selectivity for weak acids, (ii) allow for symport and channel modes of permeation, and thereby (iii) avoid proton leakage via a Grotthuss mechanism. This function is enabled without the need for a major conformational transition, but simply by modifying the free-energy landscape of the substrate using the involved proton. Further computational studies should be directed towards elucidating the details of the coupled process of histidine protonation and anion binding, as well as the fate of the proton on substrate exit, if possible by using reactive H^+^ models.

## Methods

### Structure preparation

The FNT structures were obtained from the Protein Data Bank^39^ with the following PDB IDs: 4FC4 (NirC, chains A-E), 3TDO (HSC), 3KCU (*Ec*FocA), and 3KLY (*Vc*FocA). All detergent fragments were removed, while water molecules were kept. The missing Glu23 side chain in the *Vc*FocA structure was added using the WhatIf web server (http://swift.cmbi.ru.nl/servers/html/index.html). The missing Met1 residue in chain D of the NirC structure was added with PyMol^40^. All incomplete termini, including the termini at the missing loop in the *Ec*FocA structure, were capped with an acetyl or N-methyl group.

### Simulation setup and parameters

The simulations of all investigated FNTs were set up as follows: The pentamer was embedded in a POPC (1-palmitoyl-2-oleoyl-sn-glycero-3-phosphocholine) lipid bilayer (253 lipid molecules) with g_membed^41^, in a simulation box shaped as a hexagonal prism with volume of ~1400 nm^3^. The system was solvated with ~31000 TIP3P water molecules^42^, and neutralised with the appropriate number of Cl^−^ counterions. For the computational electrophysiology (CompEl) simulations^32^, 1 M or 200 mM NaCl or NaNO_2_ were added, and the system was duplicated, translated along the *z*-axis (normal to the membrane), and stitched to the single system to yield a double membrane system. In the CompEl simulations, 2 (NirC) or 3 (*Vc*FocA) POPC molecules were placed in the central cavity among the five monomers (as suggested by the electron density features identified in the crystal structures^19,24^), in order to prevent possible leak permeation events. Typical simulation boxes of the single- and double-membrane systems (used for umbrella sampling and CompEl simulations, respectively) are shown in Supplementary Fig. S7.

For both, equilibrium and CompEl simulations, the potential energy of the system was first minimised, after which the water and lipid molecules were equilibrated for minimum 20 ns, while restraining the positions of all heavy atoms of the protein with reference to the crystal structure. Then, free unrestrained simulations of minimum 150 ns were conducted, and used for generating starting structures for the umbrella sampling. In the CompEl simulations, charge imbalance of 4*e*–16*e* between the two bulk compartments was introduced, leading to transmembrane potential between 120 and 1030 mV. Multiple CompEl simulation runs were performed for each tested protein/salt/salt concentration/charge imbalance combination for minimum 480 ns, and the first 50 ns from each run were discarded for equilibration. The transmembrane potential was calculated with g_potential^43^.

The MD simulations were performed with the Gromacs simulation software^43^. Bonds and angles in the water molecules were constrained using the SETTLE algorithm^44^, and the rest of the bonds in the system were constrained with the LINCS algorithm^45^. Hydrogen atoms were modelled as virtual sites, allowing for an integration step of 4 fs. Short-range repulsive and attractive dispersion interactions were described together by a Lennard-Jones (LJ) potential, which was cut off at 1.0 nm. Electrostatics were treated with the particle-mesh Ewald scheme^46,47^ using a grid spacing of 0.12 nm and a real-space cut-off of 1.0 nm. The temperature was kept at 300 K using velocity rescaling^48^ (*τ* = 2.5 ps), or, during umbrella sampling, using a stochastic dynamics integrator^49^ (*τ* = 0.5 ps). The pressure was controlled at 1 bar by a semi-isotropic coupling to a Berendsen barostat^50^ (*τ* = 2 ps) in the restrained simulations, and to a Parrinello-Rahman barostat^51,52^ (*τ* = 5 ps) in all other simulations, whereby the scaling in the *xy*-plane (membrane plane) was independent from the scaling in the *z*-direction. In the umbrella sampling simulations, the compressibility of the box in the *z*-direction was turned off, in order to avoid artefacts in the umbrella histograms.

The Amber-99SB*-ILDN force field^53,54^ for proteins, in combination with the Berger lipid parameters^55,56^, was used. The parameters of the substrates (formic acid, formate ion, nitrous acid, and nitrite ion) were derived using the Antechamber module^57^ from the AmberTools (release 1.4) package (for details see the next paragraph). The parameters for the hydronium ion were taken from ref.^58^, modified to exclude bond and angle vibrations in order to allow for an integration step of 4 fs.

#### Small molecule parametrisation

The general Amber force field (GAFF)^59^ atom types were used, due to the compatibility with the used Amber protein force field. HF/6–31* RESP charges were used, consistent with common GAFF parametrisations. Here, calculation of the electrostatic potential was performed with Gaussian^60^, while the RESP charge fit was performed by Antechamber. The topology files were created by the tleap module in AmberTools, and converted into Gromacs format by ACPYPE^61^. The partial charges of the parametrised molecules are shown in Supplementary Table S3.

The parameters were validated by calculation of the free energy of hydration of these molecules (Supplementary Table S4), where we find an agreement with experimentally obtained values within a few kJ mol^−1^ for the neutral substrates, and within ~15 kJ mol^−1^ for the ions. The free energy of hydration was calculated using the discrete thermodynamic integration method, where the coupling parameter *λ* was set to represent the interactions between the solute and water molecules. The LJ and Coulomb interactions were treated separately, such that the Coulomb interactions were turned off linearly over 5 *λ* points, while soft-core potential was employed when turning off the LJ interactions over 21 *λ* points. Each *λ* point was run for 500 ps, and the first 50 ps were discarded for equilibration. A cubic simulation box with edge of 50 Å was used, however, different sizes were tested (30–60 Å) and found to have no significant effect on the calculations. In order to be directly comparable to experimental values, the calculated free energies of hydration of the anions were corrected as previously suggested^62^. With the current setup, we found only correction type C1 to have a significant effect, and was estimated to be 69 kJ mol^−1^ for anions solvated in TIP3P water.

It has been shown that certain force field parameter sets may misrepresent close-range ion interactions leading to anion-cation overbinding^63,64^. To exclude that the strong binding of formate and nitrite to the imidazolium moiety from the central histidine is such a force field artefact, we computed the potential energy between these ions pairs and compared the results to high-level quantum mechanical (QM) calculations. To this end, we performed potential energy scans as a function of the distance between the H_*δ*_ atom of a 4-methylimidazolium cation (4MEI, used as a surrogate for the histidine side-chain) and the carbon/nitrogen atom from the formate/nitrite anion, at both, force field and QM levels of description (Supplementary Fig. S8). The QM calculations were performed at the SCS-MP2/aug-cc-VTZ^65–67^ level. The relatively large basis set was used to reduce any possible basis set superposition error. At all steps in the scan, the distance between the N_*δ*_ and H_*δ*_ atoms from the imidazolium ring was kept constrained, in order to avoid jumping of the proton to the anion. Furthermore, the angle between the 4MEI N_*δ*_ and H_*δ*_ atoms, and the C/N atom from the formate/nitrite ion was constrained at ≈180° in order to ensure a separation of the molecules, and not just a change of orientation. The calculations were performed with Orca^68^. The optimised structures from each step of the scan were further used for energy calculations at the force field level. The 4-methylimidazolium parameters were adapted from the histidine parameters from the Amber-99SB*-ILDN force field, such that no changes were done on the imidazolium moiety. The energy of the structures was first minimised with the steepest descent algorithm within 500 steps, to allow for relaxation of the bonded terms. During minimisation, the H_*δ*_ - C/N distance was constrained to the initial value, and the N_*δ*_ - H_*δ*_ - C/N angle was constrained to ≈180°, in order to prevent major deviations from the orientation between the ions in the QM optimised structures. Finally, the potential energy was estimated without periodic boundary conditions and without cutoffs for the Coulomb and LJ interactions. The results strongly suggest that the MD parameters rather underestimate than overestimate the anion-imidazolium interactions. Hence, the strong binding of the anions to the imidazolium moiety of the central histidine is not overestimated by the force field.

### PMFs for substrate permeation

The reaction coordinate for the permeation PMFs (Fig. 2) was defined as the position of the centre of mass of the permeating substrate along the *z*-axis (normal to the membrane), with respect to the centre of mass of the transmembrane part of the pentamer. The PMFs were computed using umbrella sampling^69^. In order to save computational resources, umbrella windows in different monomers and at different *z*-positions were simulated simultaneously. The permeating substrates were inserted into the pore at the respective umbrella centres as follows: neutral substrates were inserted at identical *z*-positions in each monomer, such that the distance between two windows in a pore was 1.5 nm (Supplementary Fig. S9a). To ensure that the PMFs for ions would not be biased by ion-ion interactions, ions were inserted in only one monomer per *z*-position, such that the distance between two windows in one monomer was 3.5 nm, and the minimum distance between any two permeating ions was 2.9 nm (Supplementary Fig. S9b). Water molecules that overlapped with the inserted substrates were removed, and the system was neutralised as necessary. Overlaps of the inserted substrates with protein atoms were removed by gradually switching on the LJ interactions of the substrate within 500 steps. After energy minimisation, the umbrella sampling simulations were ran for 5 ns (all simulations involving neutral substrates, and a few involving ionic substrates) or 10 ns (most of the simulations involving ionic substrates). The substrates were restrained at the umbrella centre by a harmonic potential with a force constant of 1000 and 4000 kJ mol^−1^ nm^−2^ for the neutral and ionic substrates, respectively. Additionally, the substrates were restrained to a cylinder with radius *r*_*c*_ = 0.7 nm with axis centred along the pore by applying a flat-bottomed quadratic potential in the *xy*-plane, with resulting additional force on the particle of *F*(*r*) = −*k*_*c*_(*r* − *r*_*c*_)*H*(*r* − *r*_*c*_) pointing towards the cylinder axis. Here, *r* represents the substrate distance from the cylinder axis, *k*_*c*_ = 1000 kJ mol^−1^ nm^−2^ is the force constant, and *H* is the Heaviside step function. The umbrella windows along the reaction were separated by 0.01 nm and 0.0175 nm, for the neutral and ionic substrates respectively, resulting with ~4800 and ~2700 umbrella histograms per PMF on average. A simulation time between 0.75 and 2 *μ*s was required per PMF.

The first nanosecond was removed from the umbrella sampling simulations with duration of 5 ns, and the first 3 nanoseconds were removed from the simulations with duration of 10 ns, after which the umbrella histograms were extracted. Single-channel PMFs were calculated using the periodic weighted histogram analysis method (WHAM)^70^, as implemented in the g_wham tool^71^. The integrated autocorrelation times were calculated and implemented in the WHAM procedure, with prior smoothing along the reaction coordinate with a Gaussian filter with width of 0.2 nm. The statistical error was estimated by applying the Bayesian bootstrap procedure using 50 bootstraps.

With the cylinder restraint, the umbrella simulations yield a free energy profile that corresponds to a channel density of one channel per cylinder cross-section. To obtain a profile that corresponds to a channel density of one channel per membrane cross-section occupied by one monomer, a trapezoidal correction was applied to the PMFs in the pore entrances, which reads: Δ*G*_corr_ = *k*_*B*_ *T* ln (*A*_mono_/*A*_*C*_)^72^. Here, *A*_mono_ and *A*_*C*_ denote the cross-section areas of the monomer and the cylinder, respectively, *k*_*B*_ is the Boltzmann constant, and *T* is the temperature. *A*_mono_ was estimated to be ≈10.6 nm^2^ for an FNT monomer. *A*_*C*_ was estimated from the radius *r*_*c*_ and force constant *k*_*c*_ of the flat-bottomed potential, as: *A*_*C*_ = *π*(*r*_*c*_ + 2*σ*_*c*_)^2^ = 2.01 nm^2^, where *σ*_*c*_ = (*k*_*B*_ *T*/*k*_*c*_)^1/2^ is the width of the Gaussian-shaped substrate distribution at the edge of the potential. This resulted with Δ*G*_corr_ = 4.1 kJ mol^−1^.

Finally, the average PMF Δ*G*_avg_(*z*) was calculated from the single-channel PMFs Δ*G*_*j*_(*z*) as:

1 $${e}^{-{\rm{\Delta }}{G}_{{\rm{avg}}}(z)/{k}_{B}T}={5}^{-1}\,\sum _{j=1}^{5}\,{e}^{-{\rm{\Delta }}{G}_{j}(z)/{k}_{B}T}.$$

Here, the permeation across the single channels was considered to be independent. The statistical error of Δ*G*_avg_ was calculated based on equation 1 and standard error propagation.

### Free energy of protonation

In this work, Δ*G*_prot_ was calculated using the GMCT method, which is based on a microstate description of the system and formalism in terms of electrochemical potentials, and performs Monte Carlo (MC) simulations in order to compute thermodynamic properties^33^. The microstate energy function includes contributions from the global conformation of the protein, the interaction of all titratable sites in the protein (in all possible protonation forms) with the protein “background” (the non-titratable parts of the protein) and the surrounding solution, as well as from the pair-wise interactions of all titratable sites. It also allows for inclusion of transmembrane potentials. The energy terms are pre-calculated by the GCEM module^73^ of the extended MEAD program suite (http://www.bisb.uni-bayreuth.de/People/ullmannt/index.php?name=extended-mead) using a combined molecular mechanics/continuum electrostatics model, based on the CHARMM force field and the linearised Poisson-Boltzmann equation. The GMCT calculations were performed on the NirC crystal structure. Hydrogen atoms, atomic radii, and atomic charges were added according to the CHARMM22 force field^74^. All histidine, arginine, lysine, glutamate, aspartate, cysteine, tyrosine, threonine, and serine residues were considered protonable.

#### MEAD/GCEM parameters

The solvent-accessible volume in the protein was determined with a spherical probe with radius of 1.4 Å. The membrane region was divided in three layers: membrane core with dielectric constant of 2, and polar lipid-head regions with thickness of 5 Å and a dielectric constant of 20. To ensure that our qualitative findings do not depend on the assumed dielectric constants, we tested different values for the dielectric constants of the protein (*ε* = 3, 4, 6, 10) and protein cavities (*ε* = 40, 60, 80) (Supplementary Table S2). The ionic strength of the solution was set to 150 mM, the temperature to 298.15 K, and the ion exclusion layer to 2 Å.

#### GMCT parameters

All GMCT calculations were performed with the Metropolis MC method, with temperature set to 298.15 K and interaction energy limits of 1 kcal mol^−1^ for pair moves, and 2 kcal mol^−1^ for triplet moves. The free energy perturbation method combined with the Bennet-Pande method^75^ was used, including statistical error tolerance of 0.02 kcal mol^−1^, a staging procedure with two chimeric intermediates, and multiple simulations according to the multi-move simulation scheme. Each simulation consisted of 1000 MC scans for equilibration, and another 1000 for production. The proton motive force was calculated from the transmembrane potential and the pH gradient according to ref.^73^:

2 $${\rm{proton}}\,{\rm{motive}}\,{\rm{force}}=-({k}_{B}T\,\mathrm{ln}\,10/F)\,{\rm{\Delta }}\text{pH}+{\rm{\Delta }}{\rm{\Psi }},$$

where ΔpH and ΔΨ correspond to the pH difference and the electrostatic potential difference between the cytoplasmic and the periplasmic space, and *F* is the Faraday constant.

To exclude that the qualitative findings depend on the details of the protein structure, we repeated the calculations with a protein structure from a HIS+ simulation after 500 ns of equilibration, suggesting that the protein had relaxed with respect to the histidine protonation. As expected, Δ*G*_prot_ was somewhat reduced (by ~18 kJ mol^−1^ on average), but overall, the high energetic cost for protonating the central histidine remains. In addition, we validated the large Δ*G*_prot_ estimates by GMCT using MD simulations with the technique of thermodynamic integration (TI). Accordingly, a proton was alchemically moved from the central histidine to a single histidine restrained in the bulk water, using 25 *λ*-points and 10 to 100 ns of simulation per *λ*-point. Depending on the initial coordinates, several independent TI calculations suggested Δ*G*_prot_ values around 100 kJ mol^−1^, yet with outliers in the tens of kJ mol^−1^ region, suggesting that the TI calculations were affected by very slow protein relaxations on the time scale of several hundred nanoseconds. Hence, the TI calculations do not provide fully converged Δ*G*_prot_ estimates, but they are in qualitative agreement with GMCT, providing additional evidence that the central histidine is deprotonated in the absence of an anion.

The free energy of protonation of formate in bulk solution was calculated according to the Henderson-Hasselbalch equation as ${\rm{\Delta }}{G}_{{\rm{prot}}}={k}_{B}T\,\mathrm{ln}\,{10}^{{\rm{pH}}-{\rm{p}}{K}_{a}}$, with pH = 7 and p*K*_a_ = 3.75, resulting in Δ*G*_prot_ = 18.6 kJ mol^−1^.

### PMFs for simultaneous proton/anion internalisation

The PMFs Δ*G*_sum_(*ξ*) for simultaneous internalisation of formate and hydronium ions into the NirC pore were calculated along a “sum-of-distances” reaction coordinate $\xi ={D}_{a}+{D}_{b}=\sqrt{{({{\bf{r}}}_{a}-{\bf{R}})}^{2}}+\sqrt{{({{\bf{r}}}_{b}-{\bf{R}})}^{2}}$, where **r**_*a*/*b*_ denote the centres of mass of the two ions, and **R** denotes the coordinates of the C_*ε*_ atom of the central histidine in the respective pore (Supplementary Fig. S5a). As before, the umbrella sampling technique was used. The calculations were performed with an in-house modified version of Gromacs 4. The implementation was validated using a system of non-interacting particles, whose motions are dictated only by the entropy *S*(*ξ*). This allows for an analytical solution of the free energy profile along *ξ* with respect to a reference state *ξ*_0_, such that Δ*G*_sum_(*ξ*) = −*T*Δ*S*(*ξ*), where Δ*S*(*ξ*) = *S*(*ξ*) − *S*(*ξ*_0_) = *k*_*B*_ ln [*W*(*ξ*)/*W*(*ξ*_0_)]. Here, *W*(*ξ*) denotes the number of microstates at given *ξ*, which is proportional to the product of (i) the surface of a sphere with radius *D*_*a*_ and (ii) the surface of a sphere with radius *D*_*b*_, integrated over pairs of *D*_*a*_/*D*_*b*_ that fulfil *D*_*a*_ + *D*_*b*_ = *ξ*. Hence, we have $W(\xi )\propto {\int }_{0}^{\xi }\,4\pi {D}_{a}^{2}4\pi {(\xi -{D}_{a})}^{2}\,{\rm{d}}{D}_{a}=\mathrm{(8}{\pi }^{2}{\xi }^{5})/15$. This finally yields ${\rm{\Delta }}{G}_{{\rm{sum}}}(\xi )=-{k}_{B}T\,\mathrm{ln}\,{\xi }^{5}/{\xi }_{0}^{5}$, which was perfectly matched by simulation of the test system with our modified Gromacs code.

For the protein system, one umbrella window per monomer was simulated in each umbrella sampling simulation. For each umbrella window *i*, at the beginning of the simulation the substrates were inserted in the pore at distances (*ξ*_*i*_/2) ± *l*, where *l* is a random distance between 0 and 0.5 nm, after which they were free to sample any distances *D*_*a*_ and *D*_*b*_ that fulfil the condition *ξ*_*i*_ = *D*_*a*_ + *D*_*b*_, where *ξ*_*i*_ is the respective umbrella centre. An interval of 0.04 nm between umbrella windows and a force constant of 8000 kJ mol^−1^ nm^−2^ were used, resulting with ~1500 umbrella histograms per PMF. As before, a cylindrical flat-bottomed potential was applied, such that an ion in bulk water would not miss the pore. Each umbrella sampling simulation was ran for 30 ns, thus resulting with 4.5 *μ*s simulation time per PMF.

The first 5 nanoseconds were removed from the umbrella sampling simulations for equilibration and the PMF was calculated using g_wham. The umbrella histograms from all five channels were considered in the calculation. The integrated autocorrelation times were calculated and implemented in the WHAM procedure, with prior smoothing along the reaction coordinate with a Gaussian filter with width of 0.2 nm. We estimate that these PMFs are associated with larger uncertainties as compared to the permeation PMFs, due to several contributing factors (uncertainties in the ion parameters, sampling issues, electrostatic effects, possible difference between the sampled states at low values of *ξ*). To better estimate this uncertainty, we calculated the standard error from the five single-channel PMFs. Moreover, we calculated the PMFs over different intervals of the umbrella simulations (0–10 ns, 10–20 ns, 20–30 ns) and we observe that the PMFs are reasonably converged. Therefore, for a semi-quantitative estimation of the mutual stabilisation as pursued in this study, we believe the achieved level of accuracy is appropriate.

We performed in total three sets of such umbrella sampling simulations. In one set (Supplementary Fig. S5c full line), the ions were placed from the same (periplasmic) side at the beginning of the simulations, therefore, the resulting Δ*G*_sum_(*ξ*) corresponds to the case where both ions enter the pore from the periplasmic side. In the second set (Supplementary Fig. S5c dashed line), the hydronium ion was placed from the periplasmic side, while the formate ion was placed from the cytoplasmic side at the beginning of the simulations, resulting with Δ*G*_sum_(*ξ*) that corresponds to the case where the ions enter the pore from opposite sides. In the third set (Supplementary Fig. S5c dot-dashed line), the ions were also placed at opposite sides, but such that Δ*G*_sum_(*ξ*) corresponds to a case where the hydronium ion enters from the cytoplasmic side, and the formate ion enters from the periplasmic side. For each set of umbrella sampling simulations, we also plotted “2-dimensional PMFs” along the individual *D*_*a*_ and *D*_*b*_ distances between the substrates and the central histidine (Supplementary Fig. S5e), using the information about the distance distributions contained in the simulation trajectories.

The mutual stabilisation *M* of the ions (Fig. 3) was defined as the difference between (i) the sum-of-distances PMF Δ*G*_sum_(*ξ*) (Supplementary Fig. S5c), and (ii) the sum of the single-ion PMFs for permeation Δ*G*(*z*) (Supplementary Fig. S5b), with the following adaptations. First, the PMFs for permeation of formate and hydronium ions were shifted 0.1 nm along the *z* coordinate, to account for the difference between the reference for Δ*G*(*z*) (the centre of mass of the transmembrane parts of the pentamer), and for Δ*G*_sum_(*ξ*) (the C_*ε*_ atom from the central histidine). Then, the *z* values were transformed into three-dimensional analogues *d* as $d=\sqrt{{z}^{2}+{r}^{2}}$, where *r* denotes the approximate radius of the pore. We tested values for *r* of 0, 0.15, and 0.25 nm, and we found that the value for *r* has only a small effect (Supplementary Fig. S5d). In Fig. 3 in the main text, the values of *M* taking *r* = 0.15 nm (corresponding roughly to the channel radius) are shown. The mutual stabilisation *M* was calculated as *M* = Δ*G*_sum_(*ξ*) − min_*d*_[Δ*G*_formate_(*d*) + Δ*G*_hydronium_(*ξ* − *d*)]. The statistical error was calculated by error propagation. *M* was computed for the pore region only, relative to a reference position *ξ*_0_ (*M*(*ξ*_0_) = 0), at which both ions are in one of the pore vestibules, i.e. *ξ*_0_ = |*z*_0,formate_| + |*z*_0,hydronium_|, as marked with coloured dots in Supplementary Fig. S5b,c. Therefore, Δ*G*_formate_ and Δ*G*_hydronium_ values were taken only from the PMF regions delimited by the red dots (Supplementary Fig. S5b), and such that they correspond to the direction of entrance as in the respective Δ*G*_sum_(*ξ*) used for computing *M*. Δ*G*(*d*) values at *d* < 0.3 nm were not taken into account, since at such short distances it is unclear whether a proton transfer event on the histidine already occurs.

### QM/MM calculations

QM/MM calculations were performed with the Gromacs/Orca^68^ interface. The QM region consisted of the central histidine side chain in one monomer and the bound formate/nitrite ion, and was closed by a “link atom” constructed as a virtual site by a linear combination of the histidine C_*α*_ and C_*β*_ atoms. The charge of the histidine C_*α*_ atom was smeared over the neighbouring atoms in order to prevent overpolarisation on the QM/MM border. Starting structures were taken from the end frames of the umbrella sampling simulations from the permeation PMFs at the energy minima identified in the PMF curves. After a short energy minimisation, free QM/MM simulations were performed, with the following parameters: stochastic dynamics integrator (*τ* = 0.1 ps) with an integration step of 1 fs, temperature set to 300 K, pressure controlled at 1 bar by semiisotropic coupling to the Parrinello-Rahman barostat (*τ* = 2 ps), LJ and Coulomb interactions cut-off at 2 nm, dispersion correction for the energy and pressure, water bonds and angles and all other bonds in the MM region constrained as in all MD simulations, and B3LYP/aug-cc-pVDZ^67,76^ level of theory in the QM region with electrostatic embedding into the MM region.

For the QM/MM simulations in bulk water, the starting structures were taken from an equilibrium simulation of a capped histidine residue in its positively-charged form and a bound formate or nitrite ion, in a simulation box of TIP3P water. The ion was bound to the histidine in a similar orientation as in the NirC central binding site, and the positions of the histidine H_*δ*_ atom, the nitrite N atom and one of the formate O atoms were kept restrained in order to prevent dissociation of the ion from the histidine. No position restraints were used in the QM/MM simulations, for which the same parameters were used as in the QM/MM simulations of the NirC system.

In order to investigate any possible influence of the simulations parameters, we also performed multiple test simulations on the NirC/formate system, employing varying schemes for treatment of long-range electrostatics (cut-off and reaction field), with or without stochastic dynamics, different integration steps (0.5–2 fs), and different temperature coupling schemes (velocity-rescaling and stochastic dynamics). We found the observed results to be largely independent on the tested simulation parameters. Therefore, for the production simulations, we chose the cut-off scheme for electrostatic interactions, as it is compatible with the Gromacs/Orca implementation, in combination with stochastic dynamics, as this circumvents the issue of system overheating as a consequence of truncation of the electrostatic interactions at a fixed cut-off. For both, NirC/formate and NirC/nitrite systems, we also tested different starting structures, with and without MM equilibration and energy minimisation prior to the QM/MM simulations, and found similar proton dynamics in all simulations (Supplementary Fig. S6a).

### Data availability statement

The datasets generated during and/or analysed during the current study are available from the corresponding author on reasonable request.

## Electronic supplementary material

Supplementary information

## References

[1] Waight AB, Czyzewski BK, Wang D-N (2013). Ion selectivity and gating mechanisms of FNT channels. Curr. Opin. Struct. Biol..

[2] Lü W (2013). The formate/nitrite transporter family of anion channels. Biol. Chem..

[3] Suppmann B, Sawers G (1994). Isolation and characterization of hypophosphite-resistant mutants of *Escherichia coli*: identification of the FocA protein, encoded by the *pfl* operon, as a putative formate transporter. Mol. Microbiol..

[4] White WB, Ferry JG (1992). Identification of formate dehydrogenase-specific mRNA species and nucleotide sequence of the *fdhC* gene of *Methanobacterium formicicum*. J. Bacteriol..

[5] Clegg S, Yu F, Griffiths L, Cole JA (2002). The roles of the polytopic membrane proteins NarK, NarU and NirC in *Escherichia coli* K-12: two nitrate and three nitrite transporters. Mol. Microbiol..

[6] Rexach J, Fernández E, Galván A (2000). The *Chlamydomonas reinhardtii Nar1* gene encodes a chloroplast membrane protein involved in nitrite transport. Plant Cell.

[7] Wang Y (2008). Nitrite transport is mediated by the nitrite-specific high-affinity NitA transporter and by nitrate transporters NrtA, NrtB in *Aspergillus nidulans*. Fungal Genet. Biol..

[8] Czyzewski BK, Wang D-N (2012). Identification and characterization of a bacterial hydrosulphide ion channel. Nature.

[9] Wu B (2015). Identity of a *Plasmodium* lactate/H^+^ symporter structurally unrelated to human transporters. Nat. Commun..

[10] Casal M, Cardoso H, Leão C (1996). Mechanisms regulating the transport of acetic acid in *Saccharomyces cerevisiae*. Microbiology.

[11] Mariscal V (2006). Differential regulation of the *Chlamydomonas Nar1* gene family by carbon and nitrogen. Protist.

[12] Rambow J, Wu B, Rönfeldt D, Beitz E (2014). Aquaporins with anion/monocarboxylate permeability: mechanisms, relevance for pathogen-host interactions. Front. Pharmacol..

[13] Das P, Lahiri A, Lahiri A, Chakravortty D (2009). Novel role of the nitrite transporter NirC in *Salmonella* pathogenesis: SPI2-dependent suppression of inducible nitric oxide synthase in activated macrophages. Microbiology.

[14] de Paiva JB (2015). Influence of the major nitrite transporter NirC on the virulence of a Swollen Head Syndrome avian pathogenic *E*. *coli* (APEC) strain. Vet. Microbiol..

[15] Golldack A (2017). Substrate-analogous inhibitors exert antimalarial action by targeting the *Plasmodium* lactate transporter PfFNT at nanomolar scale. PLoS Pathog..

[16] Hapuarachchi SV (2017). The malaria parasite’s lactate transporter PfFNT is the target of antiplasmodial compounds identified in whole cell phenotypic screens. PLoS Pathog..

[17] Jia W, Cole JA (2005). Nitrate and nitrite transport in *Escherichia coli*. Biochem. Soc. Trans..

[18] Jia W, Tovell N, Clegg S, Trimmer M, Cole J (2009). A single channel for nitrate uptake, nitrite export and nitrite uptake by *Escherichia coli* NarU and a role for NirC in nitrite export and uptake. Biochem. J..

[19] Lü W (2012). Structural and functional characterization of the nitrite channel NirC from *Salmonella typhimurium*. Proc. Natl. Acad. Sci. USA.

[20] Sawers RG (2005). Formate and its role in hydrogen production in *Escherichia coli*. Biochem. Soc. Trans..

[21] Beyer L (2013). Coordination of FocA and pyruvate formate-lyase synthesis in *Escherichia coli* demonstrates preferential translocation of formate over other mixed-acid fermentation products. J. Bacteriol..

[22] Hunger D, Doberenz C, Sawers RG (2014). Identification of key residues in the formate channel FocA that control import and export of formate. Biol. Chem..

[23] Wang Y (2009). Structure of the formate transporter FocA reveals a pentameric aquaporin-like channel. Nature.

[24] Waight AB, Love J, Wang D-N (2010). Structure and mechanism of a pentameric formate channel. Nat. Struct. Mol. Biol..

[25] Lü W (2011). pH-dependent gating in a FocA formate channel. Science.

[26] Saier MH (1999). Phylogenetic characterization of novel transport protein families revealed by genome analyses. Biochim. Biophys. Acta.

[27] Lü W (2012). The formate channel FocA exports the products of mixed-acid fermentation. Proc. Natl. Acad. Sci. USA.

[28] Rycovska A, Hatahet L, Fendler K, Michel H (2012). The nitrite transport protein NirC from *Salmonella typhimurium* is a nitrite/proton antiporter. Biochim. Biophys. Acta.

[29] Marchetti RV (2015). A lactate and formate transporter in the intraerythrocytic malaria parasite. Plasmodium falciparum. Nat. Commun..

[30] Wiechert M, Beitz E (2017). Mechanism of formate-nitrite transporters by dielectric shift of substrate acidity. EMBO J..

[31] Lv X, Liu H, Ke M, Gong H (2013). Exploring the pH-dependent substrate transport mechanism of FocA using molecular dynamics simulation. Biophys. J..

[32] Kutzner C, Grubmüller H, de Groot BL, Zachariae U (2011). Computational electrophysiology: the molecular dynamics of ion channel permeation and selectivity in atomistic detail. Biophys. J..

[33] Ullmann RT, Ullmann GM (2012). GMCT: A Monte Carlo simulation package for macromolecular receptors. J. Comput. Chem..

[34] Hub JS, de Groot BL (2008). Mechanism of selectivity in aquaporins and aquaglyceroporins. Proc. Natl. Acad. Sci. USA.

[35] Zifarelli G, Pusch M (2010). The role of protons in fast and slow gating of the *Torpedo* chloride channel ClC-0. Eur. Biophys. J..

[36] Feng L, Campbell EB, MacKinnon R (2012). Molecular mechanism of proton transport in CLC Cl^−^/H^+^ exchange transporters. Proc. Natl. Acad. Sci. USA.

[37] Doberenz C (2014). Pyruvate formate-lyase interacts directly with the formate channel FocA to regulate formate translocation. J. Mol. Biol..

[38] Falke D, Doberenz C, Hunger D, Sawers RG (2016). The glycyl-radical enzyme 2-ketobutyrate formate-lyase, TdcE, interacts specifically with the formate-translocating FNT-channel protein FocA. Biochem. Biophys. Rep..

[39] Berman HM (2000). The Protein Data Bank. Nucleic Acids Res..

[40] Schrödinger, LLC. The PyMOL molecular graphics system, version 1.7 (2015).

[41] Wolf MG, Hoefling M, Aponte-Santamara C, Grubmüller H, Groenhof G (2010). g_membed: Efficient insertion of a membrane protein into an equilibrated lipid bilayer with minimal perturbation. J. Comput. Chem..

[42] Jorgensen WL, Chandrasekhar J, Madura JD, Impey RW, Klein ML (1983). Comparison of simple potential functions for simulating liquid water. J. Chem. Phys..

[43] Pronk S (2013). GROMACS 4.5: a high-throughput and highly parallel open source molecular simulation toolkit. Bioinformatics.

[44] Miyamoto S, Kollman PA (1992). SETTLE: an analytical version of the SHAKE and RATTLE algorithm for rigid water models. J. Comput. Chem..

[45] Hess B, Bekker H, Berendsen HJC, Fraaije JGEM (1997). LINCS: a linear constraint solver for molecular simulations. J. Comput. Chem..

[46] Darden T, York D, Pedersen L (1993). Particle mesh Ewald: An Nlog(N) method for Ewald sums in large systems. J. Chem. Phys..

[47] Essmann U (1995). A smooth particle mesh Ewald method. J. Chem. Phys..

[48] Bussi G, Donadio D, Parrinello M (2007). Canonical sampling through velocity rescaling. J. Chem. Phys..

[49] Berendsen, H. J. C. & van Gunsteren, W. F. Practical algorithms for dynamic simulations. *Molecular*-*dynamics simulation of statistical*-*mechanical systems* 43–65 (1986).

[50] Berendsen HJC, Postma JPM, van Gunsteren WF, DiNola A, Haak JR (1984). Molecular dynamics with coupling to an external bath. J. Chem. Phys..

[51] Parrinello M, Rahman A (1981). Polymorphic transitions in single crystals: A new molecular dynamics method. J. Appl. Phys..

[52] Nosé S, Klein ML (1983). Constant pressure molecular dynamics for molecular systems. Mol. Phys..

[53] Hornak V (2006). Comparison of multiple Amber force fields and development of improved protein backbone parameters. Proteins.

[54] Lindorff-Larsen K (2010). Improved side-chain torsion potentials for the Amber ff99SB protein force field. Proteins.

[55] Berger O, Edholm O, Jähnig F (1997). Molecular dynamics simulations of a fluid bilayer of dipalmitoylphosphatidylcholine at full hydration, constant pressure, and constant temperature. Biophys. J..

[56] Tieleman DP, Berendsen HJC, Sansom MSP (1999). An alamethicin channel in a lipid bilayer: molecular dynamics simulations. Biophys. J..

[57] Wang J, Wang W, Kollman PA, Case DA (2006). Automatic atom type and bond type perception in molecular mechanical calculations. J. Mol. Graph. Model.

[58] Wolf MG, Groenhof G (2014). Explicit proton transfer in classical molecular dynamics simulations. J. Comput. Chem..

[59] Wang J, Wolf RM, Caldwell JW, Kollman PA, Case DA (2004). Development and testing of a general Amber force field. J. Comput. Chem..

[60] Frisch, M. J. *et al*. Gaussian 09 Revision A.02 (Gaussian Inc., Wallingford CT, 2009).

[61] Sousa da Silva AW, Vranken WF (2012). ACPYPE - AnteChamber PYthon Parser interfacE. BMC Res. Notes.

[62] Reif MM, Hünenberger PH (2011). Computation of methodology-independent single-ion solvation properties from molecular simulations. III. Correction terms for the solvation free energies, enthalpies, entropies, heat capacities, volumes, compressibilities, and expansivities of solvated ions. J. Chem. Phys..

[63] Venable RM, Luo Y, Gawrisch K, Roux B, Pastor RW (2013). Simulations of anionic lipid membranes: development of interaction-specific ion parameters and validation using NMR data. J. Phys. Chem. B.

[64] Yoo J, Aksimentiev A (2016). Improved parameterization of amine-carboxylate and amine-phosphate interactions for molecular dynamics simulations using the CHARMM and AMBER force fields. J. Chem. Theory Comput..

[65] Møller C, Plesset MS (1934). Note on an approximation treatment for many-electron systems. Phys. Rev..

[66] Grimme S (2003). Improved second-order Møller–Plesset perturbation theory by separate scaling of parallel- and antiparallel-spin pair correlation energies. J. Chem. Phys..

[67] Kendall RA, Dunning TH, Harrison RJ (1992). Electron affinities of the first-row atoms revisited. Systematic basis sets and wave functions. J. Chem. Phys..

[68] Neese F (2012). The ORCA program system. Wiley Interdiscip. Rev. Comput. Mol. Sci..

[69] Torrie GM, Valleau JP (1974). Monte Carlo free energy estimates using non-Boltzmann sampling: application to the sub-critical Lennard-Jones fluid. Chem. Phys. Lett..

[70] Kumar S, Rosenberg JM, Bouzida D, Swendsen RH, Kollman PA (1992). The weighted histogram analysis method for free-energy calculations on biomolecules. I. The method. J. Comput. Chem..

[71] Hub JS, de Groot BL, van der Spoel D (2010). g_wham - A free weighted histogram analysis implementation including robust error and autocorrelation estimates. J. Chem. Theory Comput..

[72] Allen TW, Andersen OS, Roux B (2006). Molecular dynamics - potential of mean force calculations as a tool for understanding ion permeation and selectivity in narrow channels. Biophys. Chem..

[73] Ullmann RT, Andrade SLA, Ullmann GM (2012). Thermodynamics of transport through the ammonium transporter Amt-1 investigated with free energy calculations. J. Phys. Chem. B.

[74] MacKerell AD (1998). All-atom empirical potential for molecular modeling and dynamics studies of proteins. J. Phys. Chem. B.

[75] Shirts MR, Bair E, Hooker G, Pande VS (2003). Equilibrium free energies from nonequilibrium measurements using maximum-likelihood methods. Phys. Rev. Lett..

[76] Stephens PJ, Devlin FJ, Chabalowski CF, Frisch MJ (1994). *Ab initio* calculation of vibrational absorption and circular dichroism spectra using density functional force fields. J. Phys. Chem..

[77] Smart OS, Neduvelil JG, Wang X, Wallace BA, Sansom MSP (1996). HOLE: a program for the analysis of the pore dimensions of ion channel structural models. J. Mol. Graph..

