# Supplementary material for: Energetics and mechanism of anion permeation across formate-nitrite transporters
Source: Sci Rep. 2017 Sep 20;7:12027. doi: 10.1038/s41598-017-11437-0 (PMC5607303; doi:10.1038/s41598-017-11437-0)
Supplement: Supplementary file 1 — Supplementary information [file 41598_2017_11437_MOESM1_ESM.pdf]

Supplementary information for Energetics and mechanism of anion permeation across formate-nitrite transporters

Kalina Atkovska[1,2] **and Jochen S. Hub**[1,2,*]

[1]University of Goettingen, Institute for Microbiology and Genetics, Goettingen, 37077, Germany
[2]University of Goettingen, Göttingen Center for Molecular Biosciences, Goettingen, 37077, Germany
[*]jhub@gwdg.de

Supplementary figures and tables

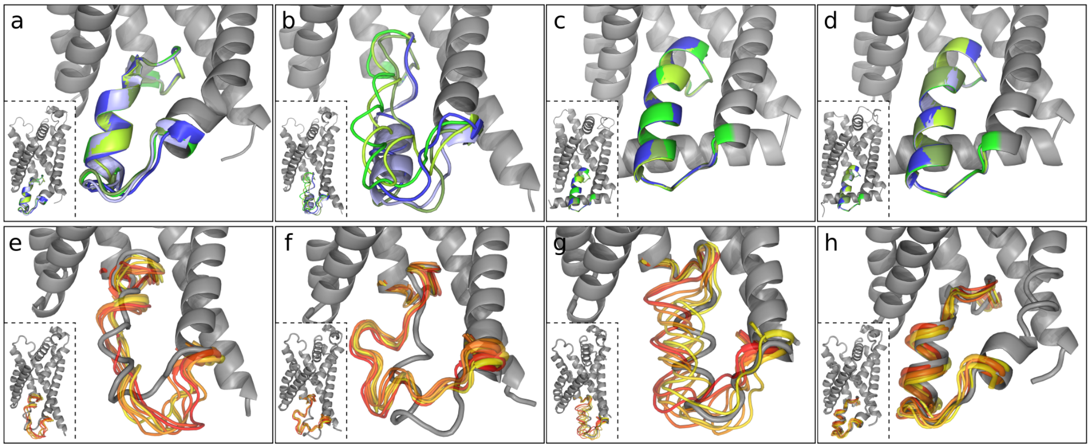

Figure S1. The region of the Ω-loop in the crystal structures (a-d) and in simulation (e-h). (a-d) One monomer shown as a grey cartoon, the region of the Ω-loop from all monomers shown as an overlay in different shades of green and blue. (a) *Ec*FocA, PDB ID: 3KCU, (b) *Vc*FocA, PDB ID: 3KLY, (c) HSC, PDB ID: 3TDO, (d) NirC, PDB ID: 4FC4. The Ω-loop exhibits different conformations in different monomers in *Vc*FocA, in contrast to the same region in the other depicted FNT structures. (e-f) The region of the Ω-loop shown as an overlay of simulation snapshots from 20 ns-intervals from a 150 ns equilibrium simulation in shades from yellow to red. The crystal structure of the respective monomer is shown as a grey cartoon. (e,f,g) Three monomers from the *Vc*FocA structure, demonstrating high degree of variability of the Ω-loop in time and among monomers. (h) A representative monomer from the *Ec*FocA structure, demonstrating stable conformation of the Ω-loop, matching the crystal structure. For orientation, the entire monomer is shown in the inset in each of the figures.

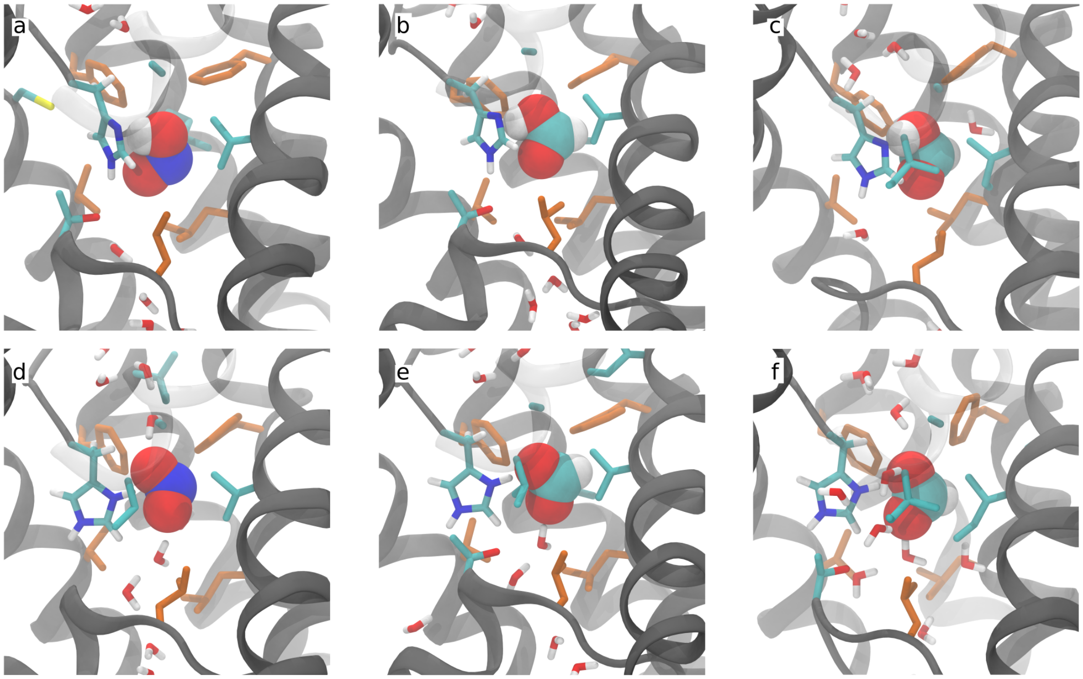

Figure S2. Simulation snapshots from the umbrella windows in the region of the central histidine shown for illustration. The permeating substrate: (a) nitrous acid, (b,c) formic acid, (d) nitrite, (e,f) formate is shown as spheres. The binding of the ions to the HIS+ pore (d,e,f) is more stable in comparison to the binding of the neutral substrates to the HIS0 pore (a,b,c), which are more dynamic in the central chamber. Only the HIS+ pore (d,e,f) is fully hydrated. The side chains of all residues with atoms within 5 Å from the substrate, and all water molecules with atoms within 10 Å from the substrate are shown as sticks. The residues forming the constriction sites are coloured orange. Only the hydrogen atoms from the permeating substrate, the central histidine and the water molecules are shown. The protein is shown as a grey cartoon, helix TM3 removed, and subhelix TM5b made transparent for clarity. (a,b) NirC HIS0, $z \approx 0.06$ nm, (c) *Vc*FocA HIS0, $z \approx -0.25$ nm, (d) NirC HIS+, $z \approx 0.23$ nm, (e) NirC HIS+, $z \approx 0.21$ nm, (f) *Vc*FocA HIS+, $z \approx -0.25$ nm.

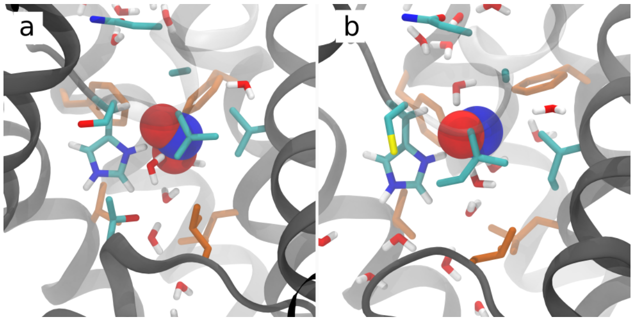

Figure S3. Simulation snapshots from the CompEl simulations in the region of the central histidine. (a) NirC and (b) *Vc*FocA shown as a grey cartoon, helix TM3 removed, and subhelix TM5b made transparent for clarity. Nitrite ion is shown as spheres. The side chains of all residues with atoms within 5 Å from the nitrite ion, and all water molecules with atoms within 10 Å from the nitrite ion are shown as sticks. The residues forming the constriction sites are coloured orange. Only the hydrogen atoms from the permeating substrate, the central histidine and the water molecules are shown. The snapshots are from simulations with 200 mM salt concentration and charge imbalance of 4e. In all CompEl simulations, ions were found to frequently enter the pore and bind to the central histidine, however, this strong binding prevented efficient translocation via the pore.

Protein	Anion	Salt conc.	t^a (μs)	ΔU^b (mV)	Φ^c (μs^{-1})	G^d (pS)
NirC	NO_2^-	1M	0.9	~ 820	0	0
NirC	NO_2^-	0.2M	1.95	~ 260	0	0
NirC	Cl^-	1M	2.23	~ 950	0.045	≈ 0
*Vc*FocA	NO_2^-	1M	1.3	~ 640	0.769	≈ 0.12
*Vc*FocA	NO_2^-	0.2M	1.95	~ 260	0	0
*Vc*FocA	Cl^-	1M	2.21	~ 720	2.217	≈ 0.31

Table S1. Anion permeation across HIS+ NirC and HIS+ *Vc*FocA from computational electrophysiology simulations. [a]Total simulation time t. [b]Average transmembrane potential ΔU from all simulation runs. [c]Single-channel flux Φ. The single channels were considered bidirectional and independent, since the few observed permeation events occurred in both directions and through different monomers. Therefore, all observed full permeations across both pentamers in all simulation runs were lumped together (N_{total}), and Φ was estimated as $\Phi = N_{\text{total}}/(10t)$. [d]Conductance G, estimated as $G = \Phi e/\Delta U$, where $e = 1.6 \times 10^{-19}$ C is the unit charge. Overall, the conductance is far lower than experimentally measured, suggesting that a knock-on mechanism does not provide the means for anion permeation across the FNTs.

$\varepsilon_{\text{protein}}$ [a]	$\varepsilon_{\text{cavity}}$ [b]	ΔG_{prot} [c] (kJ mol^{-1})
4	80	70.2 ± 3.6 (default)
4	60	72.5 ± 3.8
4	40	76.1 ± 4.4
3	80	86.5 ± 4.9
6	80	52.2 ± 2.3
10	80	36.2 ± 1.3

Table S2. Free energy of protonation ΔG_{prot} of the central histidine in NirC, as computed with GMCT, assuming different dielectric constants for the protein and the cavities. Irrespective of the dielectric constants, a large protonation free energy is predicted for the histidine, suggesting that the histidine is mostly deprotonated in absence of an anion. [a]Dielectric constant of the protein and of the [b]protein cavities (default $\varepsilon_{\text{protein}} = 4$, $\varepsilon_{\text{cavity}} = 80$). [c]Mean and s.d. over five chains.

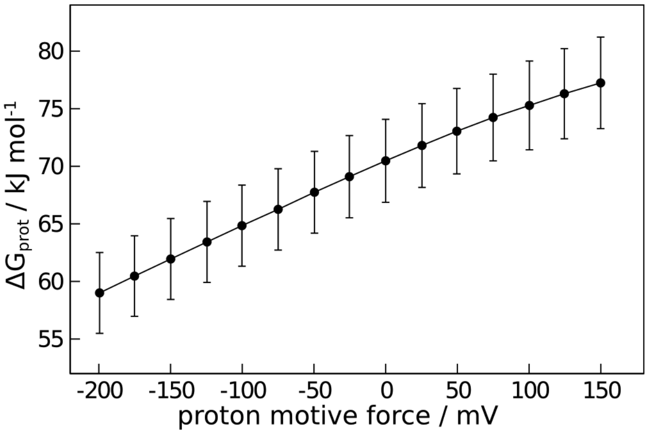

Figure S4. Free energy of protonation ΔG_{prot} of the central histidine in NirC (average and s.d. over five chains), at a proton motive force from -200 to 150 mV. Irrespective of the proton motive force, ΔG_{prot} is large, suggesting that only a marginal fraction of this residue would be doubly-protonated in absence of an anion.

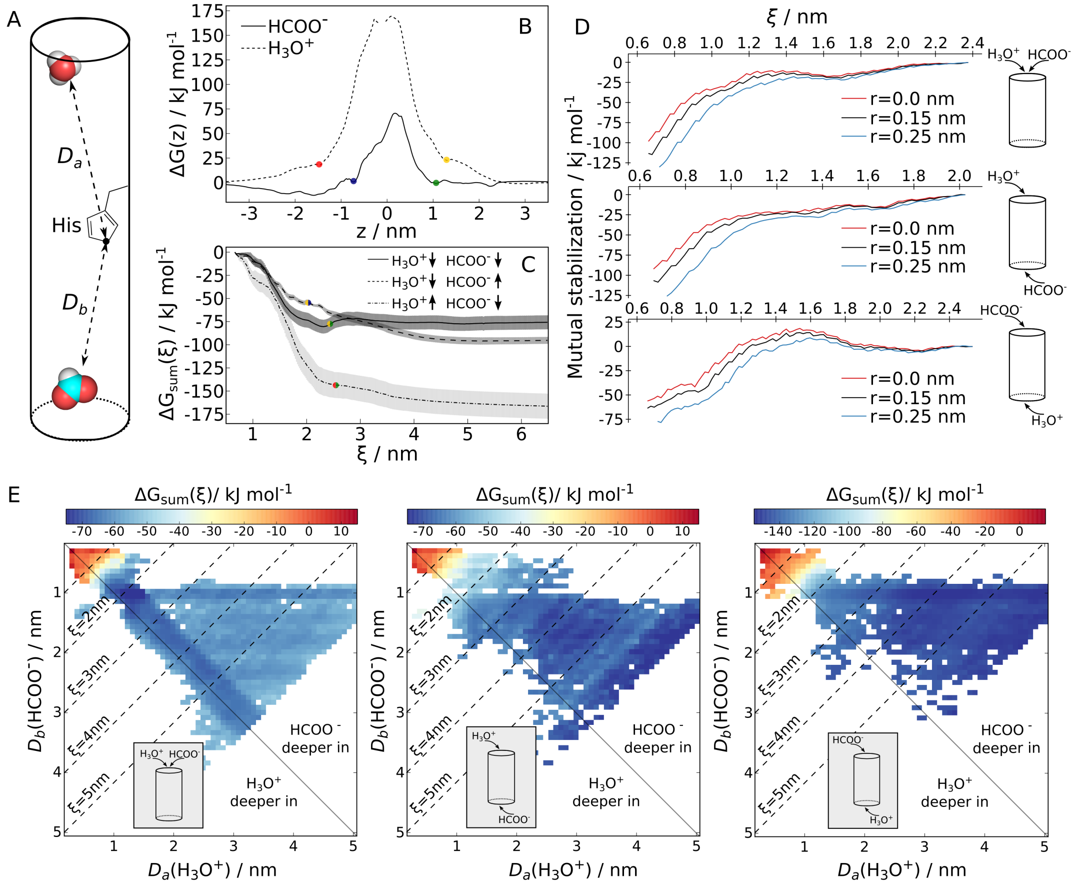

Figure S5. Mutual stabilisation of formate and hydronium ion in the NirC pore. (a) Schematic representation of the reaction coordinate $\xi = D_a + D_b$, where D_a and D_b denote the distances of the centres of mass of the investigated particles (here, hydronium and formate ions, shown as spheres) to the C_ε atom of the central histidine. (b) PMFs $\Delta G(z)$ for permeation of the formate ion (solid line) and of a classical hydronium model (dashed line) across the NirC HIS0 pore, used for calculation of the mutual stabilisation M. The reference positions used to define ξ_0 are marked as coloured dots. (c) Sum-of-distances PMFs $\Delta G_{\text{sum}}(\xi)$ for simultaneous internalisation of formate and hydronium into the NirC HIS0 pore. The arrows in the legend denote the direction of entrance of the respective ion: (↓) from the periplasmic side, (↑) from the cytoplasmic side. Standard error calculated from 5 single-channel PMFs. The dots denote ξ_0, with matching colours to the ions' direction of entrance as in (b). Note that the PMFs can not be directly compared, as the states at small sum of distances ($\xi \approx 0.7$), as well as at large sum of distances ($\xi > 3$) do not correspond to the same thermodynamic states among the three PMFs, thus rationalizing the free energy offset: At small ξ the location of hydronium differs between the PMFs (solid and dashed lines vs. dot-dashed line); at large ξ, the anion typically remained bound to the protein due to electrostatic interactions, suggesting that such positions do *not* correspond to states of bulk water. (d) Mutual stabilisation M of formate and hydronium into the NirC HIS0 pore, considering different values of r (for details see Methods). (e) 2-dimensional profiles along the individual D_a and D_b distances between the ions and the central histidine, extracted from the umbrella sampling simulations used for computing $\Delta G_{\text{sum}}(\xi)$. The top-left to bottom-right diagonal denotes points where both ions are at equal distance to the central histidine. Top to left diagonals (some shown as dashed lines) denote points with equal value of ξ. The formate ion tends to enter the pore first.

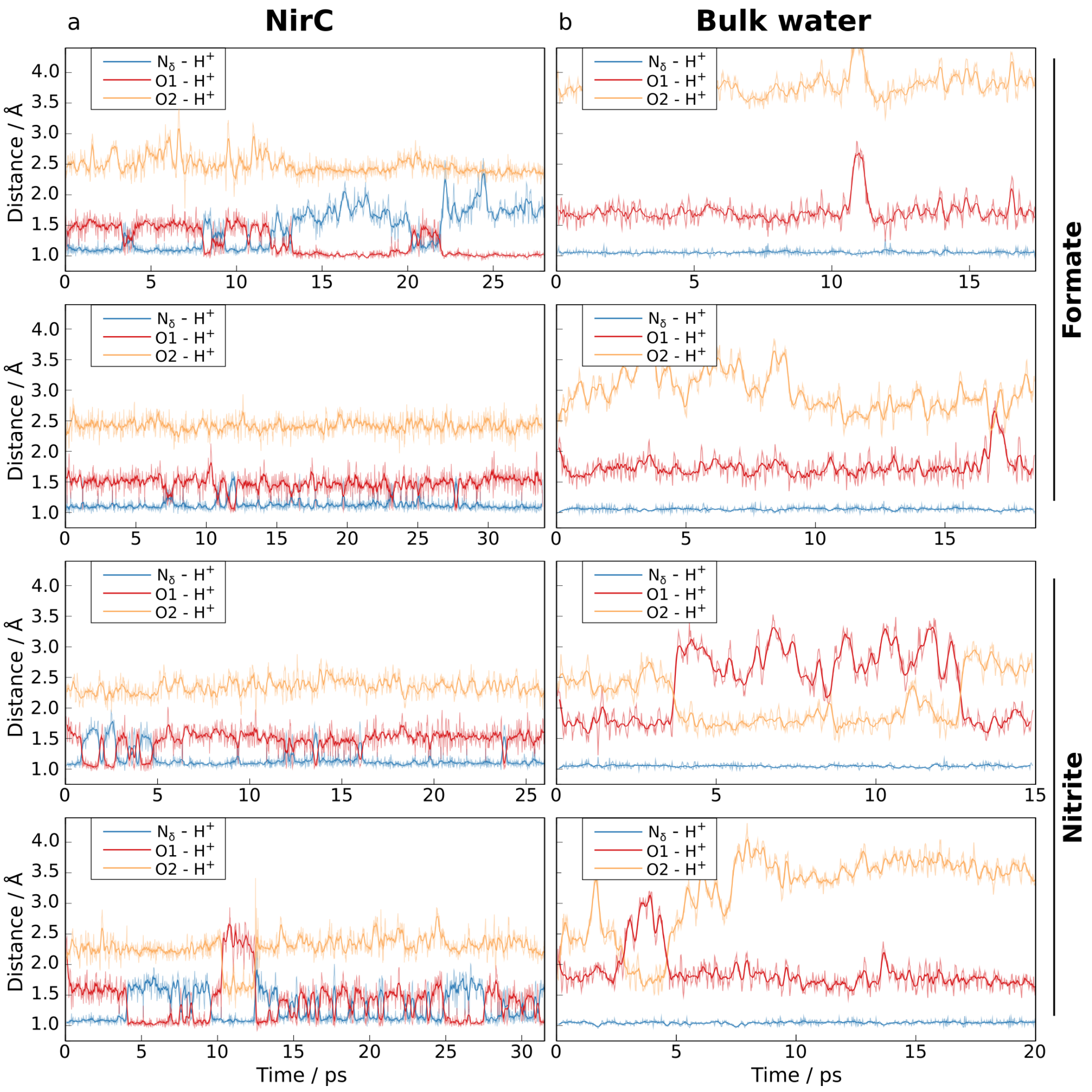

Figure S6. Multiple QM/MM simulations of proton transfer between a histidine side chain and formate (top two rows) or nitrite (bottom two rows). The simulations were either conducted in NirC involving the central histidine (left column) or in bulk water (right column). The curves present the distances (raw traces and running averages) of the jumping proton to the N_δ atom from the histidine (blue) and to the two oxygen atoms from the formate and nitrite ions (red and orange). In bulk water, the proton remains bound to the histidine within multiple ~ 20 ps simulations, as expected from the higher pK_a value of histidine (right column). The more hydrophobic protein channel environment favours the neutral species, thus rationalising proton transfer between the histidine side chain and the formate/nitrite ion on a picosecond time scale (left column).

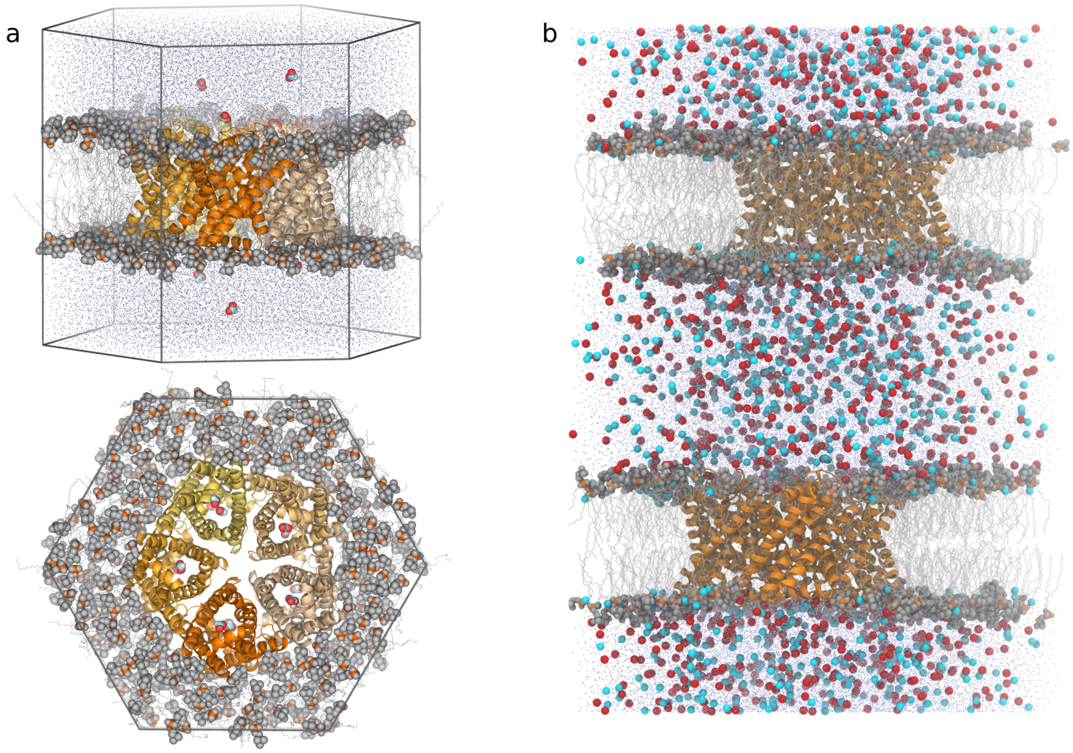

Figure S7. Typical simulation systems for the umbrella sampling simulations (a) and the CompEl simulations (b). Shown are: the protein as a cartoon, lipid heads as spheres (with phosphorus atoms coloured orange), lipid tails as lines, water oxygens as blue dots, and (a) formate ions as spheres coloured by atom, or (b) sodium and chloride ions as spheres coloured blue and red, respectively. Depicted in (a) are a side view of the box, with a slight tilt, and a top view of the box with omitted water molecules, while (b) depicts the side view of the box. The protein chains are coloured in different shades of orange in (a).

formic acid		nitrous acid		formate ion		nitrite ion	
H1	0.045999	O1	-0.132386	C1	0.849269	O1	-0.475567
C1	0.710562	N1	0.094006	O1	-0.825141	N1	-0.048866
O1	-0.578108	O2	-0.346016	O2	-0.825141	O2	-0.475567
O2	-0.650276	H1	0.384396	H1	-0.198987		
H2	0.471822						

Table S3. Partial charges of the parameterised small molecules.

substrate	Calculated	Experimental	Exp.-Calc.
formic acid	-27.7 ± 0.4	-29.3[a]	-1.6
nitrous acid	-15.9 ± 0.4	-9.6[b]	6.3
formate	-334 ± 0.7	-319[a]	15
nitrite	-321 ± 0.6	-305[c]	16

Table S4. Hydration free energies of the parameterised molecules in kJ mol^{-1}. Experimental values from references 1[a], 2[b], and 3[c].

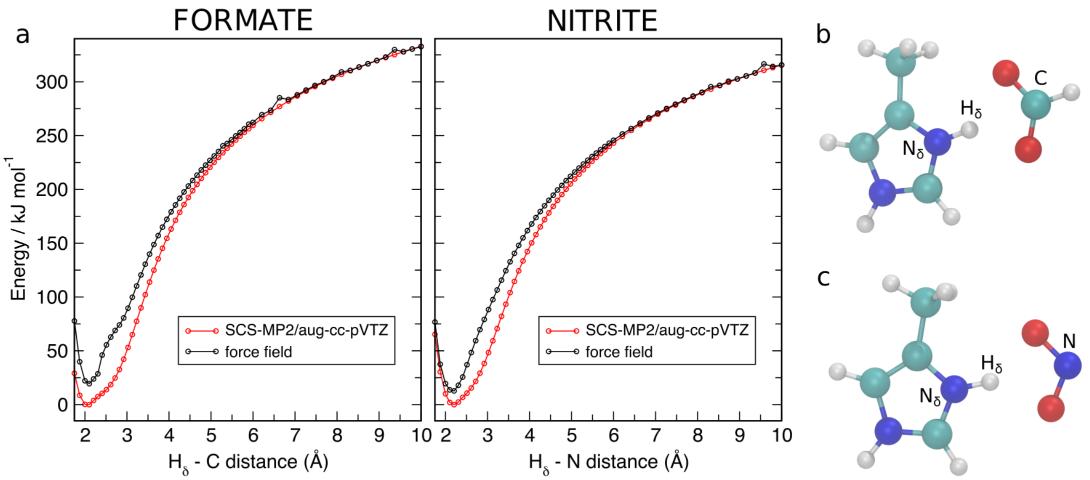

Figure S8. (a) Potential energy of the 4-methylimidazolium/formate (left) and 4-methylimidazolium/nitrite (right) ion pairs in vacuum as a function of distance between the H_δ atom from the imidazolium moiety and the carbon/nitrogen atom from the formate/nitrite ion. The QM calculations were performed at the SCS-MP2/auc-cc-pVTZ level. The minima of the QM curves were shifted to 0 kJ mol^{-1}, and the force field curves were aligned to the QM curves to the right. There is no overbinding between the ions as compared to the QM calculations. (b/c) The 4-methylimidazolium/formate (b) and 4-methylimidazolium/nitrite (c) ion pairs shown as ball-and-stick representation.

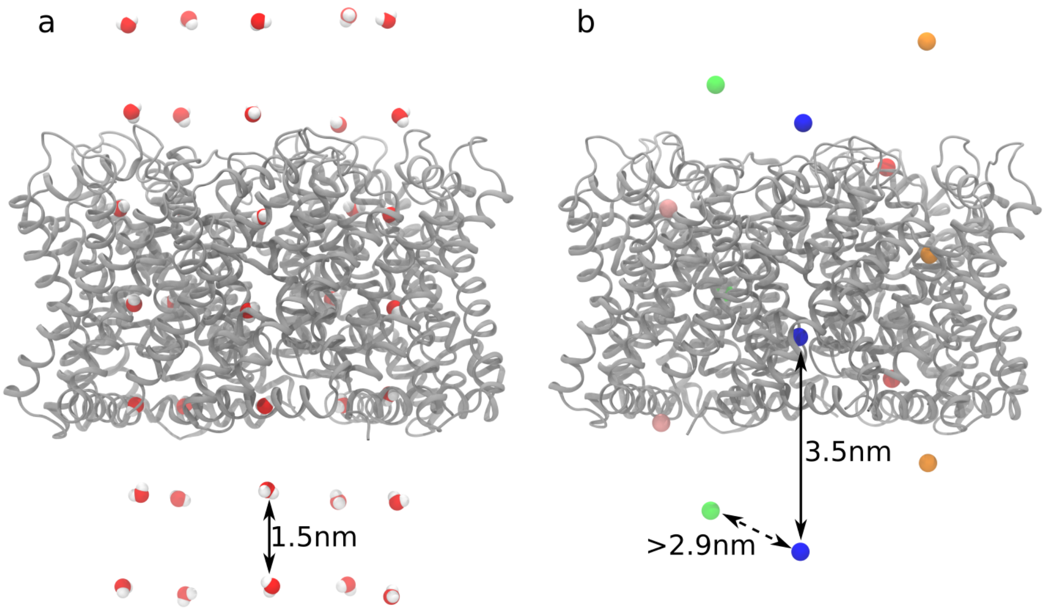

Figure S9. Placement of neutral substrates (a) and ions (b) in the umbrella sampling simulations for calculation of the permeation PMFs. The protein is shown as a cartoon, water molecules in (a) and chloride ions in (b) are shown as spheres. The neutral substrates are placed at equal *z*-position in each monomer, such that the distance between substrates in one monomer is 1.5 nm. The chloride ions are colour-coded by their association to different monomers, and they are placed such that the distance between two ions in one monomer is 3.5 nm, and the minimum distance between ions from different monomers is 2.9 nm, to ensure a minimal (if any) effect of the ion-ion interactions on the PMFs.

References

1. Kelly, C. P., Cramer, C. J. & Truhlar, D. G. Aqueous solvation free energies of ions and ion-water clusters based on an accurate value for the absolute aqueous solvation free energy of the proton. *J Phys Chem B* **110**, 16066–81 (2006). DOI 10.1021/jp063552y.
2. Becker, K. H., Kleffmann, J., Kurtenbach, R. & Wiesen, P. Solubility of nitrous acid (HONO) in sulfuric acid solutions. *J Phys Chem* **100**, 14984–14990 (1996).
3. Florián, J. & Warshel, A. Langevin dipoles model for *ab initio* calculations of chemical processes in solution: Parametrization and application to hydration free energies of neutral and ionic solutes and conformational analysis in aqueous solution. *J Phys Chem B* **101**, 5583–5595 (1997).
